# The complexity of caregiving amid challenges: experiences of families with young caregivers

**DOI:** 10.1590/1980-220X-REEUSP-2025-0444en

**Published:** 2026-05-11

**Authors:** Gabrieli Patricio Rissi, Bianca Machado Cruz Shibukawa, Camila Moraes Garollo Piran, Beatriz Sousa da Fonseca, Alana Vitória Escritori Cargnin, Maycon Hoffmann Cheffer, Ieda Harumi Higarashi

**Affiliations:** 1 Universidade Estadual de Maringá, Programa de Pós-Graduação em Enfermagem, Maringá, PR, Brazil.; 2 Universidade Federal de Mato Grosso do Sul, Departamento de Enfermagem, Três Lagoas, MS, Brazil.; 3 Politécnico de Leiria, Leiria, Portugal.; 4 Universidade Estadual do Centro Oeste, Departamento de Enfermagem, Guarapuava, PR, Brazil.

**Keywords:** Caregivers, Child, Adolescent, Caregiver Burden, Family Relations

## Abstract

**Objective:**

To build a substantive theoretical model on the trajectory of families with young caregivers in the process of caring for a family member with a disabling condition.

**Methodology:**

Exploratory, qualitative study, conducted using the references of Grounded Theory and Complex Thinking. Data collection took place between August 2022 and October 2023, in the homes of 15 young people and 7 family caregivers. The analysis followed three stages: open, axial, and selective coding, with the support of ATLAS.ti^®^ software.

**Results:**

The trajectory of families with young caregivers proved to be a process permeated by barriers to care, relational and ethical challenges, and multiple repercussions for young people and caregivers. Barriers associated with family unavailability and care overload were evident, as were challenges linked to the resistance of the family member being cared for, fear of reporting, and concern for the young caregiver’s quality of life, as well as consequences that impact the well-being of young people and their guardians.

**Conclusion:**

Care provided by young people is a complex, multifactorial, and dynamic phenomenon that requires attention from health professionals, especially in primary care, based on a comprehensive understanding of the contexts and social determinants.

## INTRODUCTION

In the modern world, where all people in good health and of working age are absorbed into the labor market, the responsibility of caregiving often ends up being assigned to those who remain at home. In this context, the figure of young caregivers emerges; this group is made up of individuals under the age of 18 who perform the function of informal and unpaid care, assistance, or support to family members^(1)^. Due to the complexity of the situation, the prevalence of young caregivers worldwide is uncertain, but it is estimated to be between 2 and 8% and growing^(2)^.

It should also be noted that adolescents are individuals in a phase of physiological, cognitive, and psychological maturation and are therefore not in an ideal position to take on such responsibility. However, households in which young caregivers need to take on caregiving responsibilities usually have a family configuration in which only the caregiver and the care recipient remain at home, since the other family members have other activities outside the home^(3)^.

The family’s choice of a young caregiver is sometimes a natural decision to maintain the functioning of family dynamics. For young people, however, there is rarely a choice or option not to take on such a responsibility; they are simply tasked with performing the necessary activities. By taking on such a commitment, even if involuntarily, various disruptions are generated in their daily lives as a result of the care provided^(2)^.

Although there is already some evidence of the deterioration of health, well-being, and family relationships among young caregivers, these issues remain hidden in the agendas of health care discussions and public policies in the area. Transformation in the field of health care requires a learning environment that adopts a perspective of complexity^(4)^.

In this sense, complex thinking, which emphasizes the interconnection and interdependence of all phenomena, can explain this situation, not as an isolated problem, but as part of a complex system of social and economic relations^(5)^. Understanding the trajectory of families with young caregivers in the process of caring for a family member would allow health professionals to gain a broader understanding of the phenomenon, while also helping to share the burden of care^(6)^.

Therefore, this study is justified based on the importance of seeking to reveal the role of young caregivers and their families, recognizing them as beings with rights, in search of guaranteed access to health, the promotion of a healthy adolescence, and the construction of an adequate family dynamic^(2)^. Given the above, the objective was to construct a substantive theoretical model on the trajectory of families with young caregivers in the process of caring for a family member with a disabling condition. In this context, the research question was defined as: how is the trajectory of families with young caregivers in the process of caring for a family member with a disabling condition configred?

## METHOD

### Study Design

Exploratory, qualitative research using Complex Thinking^(5)^ as a theoretical framework and Grounded Theory (GT), in the Straussian strand^(7)^, as a methodological framework.

The study setting corresponded to the homes of young people and family caregivers residing in a municipality located in northwestern Paraná, Brazil.

### Location, Population, and Selection Criteria

The information was collected between August 2022 and October 2023, using sociodemographic questionnaires formulated by the researcher himself and conducting semi-structured face-to-face interviews. Unstructured observations were also used, but conducted according to a standardized methodology, with notes in a field diary to ensure uniformity in the records.

Prior to the data acquisition process, the collection instruments underwent theoretical content validation. This process involved consulting three specialists in the field of child and adolescent health, which was relevant to ensure the accuracy and relevance of the tools. No pilot test was conducted, considering that, in line with the qualitative approach adopted, the instruments were designed to be flexible and adjustable throughout the data collection and analysis process. The interviews were audio-recorded and transcribed, lasting an average of 30 minutes. Subsequently, the full records of the interviews underwent a minimal process of linguistic standardization.

Thus, for the first sample group, composed of 15 young caregivers and chosen because they were the main object of the study, the selection criteria were: being a child or adolescent under 18 years of age who provides care, assistance, or support to a family member dependent on care; performing activities such as health care, assistance with daily activities, domestic and/or socio-emotional assistance; and not being a mother or father responsible for the care of minor children.

In order to identify young caregivers, the study contacted Community Health Workers (CHWs) from each Basic Health Unit (BHU) and Family Health Support Unit (FHSU) in the municipality, totaling 37 health services. In addition, those in charge of the Home Care Service (HCS) were also approached. After the CHW and HCS teams identified potential participants, the researchers contacted the families directly, presenting the study’s objectives and inviting the young people and their legal guardians to participate voluntarily.

Subsequently, it was necessary to conduct a more detailed analysis of the phenomenon under study with the family members, who were primarily responsible for the care of the young caregivers. For them, the inclusion criteria were: being the legal guardian of the young caregiver and being the care coordinator within the family.

Thus, seven family members made up the second sample group, comprising eight of the young participants, since two were siblings and had the same family bond. Although theoretical saturation was achieved with those who were part of this group, it is evident that the other guardians of the young caregivers invited to participate in the study were unable to join the group because they refused to participate in the research or had changed their address at the time of the approach.

### Data Analysis

As recommended by Grounded Theory, in accordance with the principle of constant comparative analysis, data analysis was performed concurrently with each interview. Data collection was performed exclusively by the principal investigator, who was responsible for the study as part of her doctoral thesis and who had prior training and experience in qualitative research, as well as theoretical and methodological expertise and a review of the literature on the subject.

The interviews were conducted using semi-structured scripts, which were different for each sample group, considering the specificities of the young caregivers and their guardians. The questions were progressively adjusted throughout the collection process, in line with the preliminary analysis of the data and the progress of the theoretical sampling. The interviews were fully transcribed and checked by the researcher herself, who listened carefully to the audio recordings and field notes. The transcripts were not returned to the participants for validation, as the researcher was present at all stages of the process.

It should be noted that the inclusion of the second sample group was guided by analytical findings emerging from the first group, which highlighted the centrality of parents/guardians, family dynamics, and working conditions in the care process, requiring further theoretical exploration of these elements for the development of the substantive model.

Regarding the analytical process, open, axial, and selective coding was performed. Initially, open codes were established based on the reading of transcripts derived from the interviews. Then, in axial coding, the open codes were grouped by similarity and generated categories and subcategories. Finally, in selective coding, the categories were refined and the central phenomenon was structured.

Memos and diagrams were used throughout the analytical process, especially during open coding. To optimize and organize data analysis, ATLAS.ti^®^ software was used as a data systematization mechanism.

It should also be noted that the theoretical model constructed was validated by four participants, two young caregivers and two family members. In this process, it was found that there was no need to make changes to the model created, since most individuals were able to identify with what was proposed. It should also be noted that this study followed all the guidelines of the Consolidated Criteria for Reporting Qualitative Research (COREQ).

### Ethical Aspects

The study was approved by the Standing Committee on Ethics in Research with Human Subjects of the State University of Maringá, under opinion No. 5,707,802 and CAAE 61476022.1.0000.0104. The subjects expressed their agreement in the Terms of Consent and Free and Informed Consent, in two copies of equal content, being categorized as “young caregiver” or “family member,” followed by the number representing the order in which the interviews were conducted. In cases involving minors, the Free and Informed Consent Form was signed by their legal guardians, and the Assent Form was signed by the young caregivers. It should be noted that this study presents a snapshot of the substantive theory constructed in the context of a doctoral thesis entitled “Young caregivers and the role of Primary Health Care,” contemplating analytical fragments of the central phenomenon.

## RESULTS

### Characterization of Participants

The phenomenon under study encompassed 22 individuals, divided into two sample groups, the first consisting of 15 young caregivers and the second comprising seven family members legally responsible for the young people.

The young caregivers ranged in age from 10 to 17 years, with an average age of 13 years. Most participants were female (n = 8), brown-skinned (n = 10), and enrolled in elementary school (n = 13).

The predominant degree of kinship of the individuals under care was sibling (n = 7), followed by parents and grandparents/great-grandparents (n = 4 each). The reasons that triggered the need for family care were varied, involving chronic, congenital, and mental illnesses, as well as accidents and physical-cognitive immaturity.

Similarly, family members were predominantly female (n = 5), brown in race/color (n = 5), and aged between 40 and 49 years (n = 3). The prevailing per capita income was up to ½ minimum wage (n = 4), with the majority not employed at the time of the survey (n = 5). Regarding health status, five family members had some health condition, with chronic diseases being the most commonly reported (n = 4).

### Trajectory of Families with Young Caregivers in the Process of Caring for A Family Member with A Disabling Condition

The results showed that the need to care for a family member, particularly in situations of illness, resulting in the individual’s inability to meet their basic daily needs, is often accompanied by a series of obstacles. Among these, the most notable are family unavailability, linked to the financial context and the inevitability of work, and family overload derived from the pathological process inherent to the individual being cared for.

This context results in several challenges for the responsible family member, given that, in addition to having intrinsic concerns arising from the reality experienced, they also assume a central role as a reference for both the family member under their care and the young caregiver, taking responsibility for the entire scenario involved.

In this study, it was possible to observe that both the responsible family member and the young caregiver encountered difficulties in dealing with the illness/condition of the family member being cared for. These difficulties stemmed both from the challenges in developing the skills necessary to perform caregiving activities and from the emotional preparation required to deal with the repercussions of the illness on the physical and cognitive health of the loved one. In addition, other difficulties in providing care were relational in nature, triggered by the opposing attitudes of the family member being cared for, either through denial of the diagnosis or reluctance to accept help from family members in self-care.

The challenges faced by responsible family members related to the need for young people to care for family members did not go unnoticed either. In this regard, the presence of concerns on the part of the responsible person in relation to the young caregiver was verified; a certain compromise in the quality of life and well-being of the young person was noticeable, resulting from the overload of care activities and family absence. Furthermore, there was notable concern about the fear of reports due to the discovery of the young caregiver, as many feared society’s judgment of this practice as something illegal.

With regard to young caregivers, in turn, there was evidence of a considerable burden, as they had to manage numerous concerns, which had negative consequences for both their physical and mental health. Thus, it was possible to evidence the compromise of these caregivers’ leisure time and social life, which led them to experience significant emotional stress.

Furthermore, it was perceived that the responsible family members faced important personal dilemmas, while the young caregivers had their educational lives compromised as a result of caregiving. Despite this, the results indicated that this situation led to the development of issues related to the spirituality of the responsible family members, as many mentioned greater involvement with religious institutions and expressions of faith in God.

**Figure 1:**
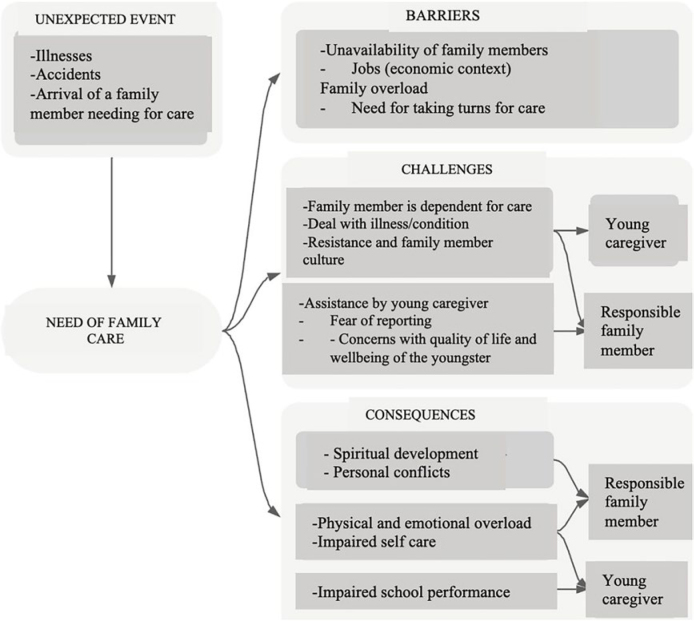
Fragments of substantive theory for the central phenomenon of the study.

Based on this analysis, fragments of substantive theory were developed for the central phenomenon: “Trajectory of families with young caregivers in the process of caring for a family member with a disabling condition,” represented in Figure 1.

### Portraits of Care: Family Challenges

It was evident that both family members managing care and young caregivers have numerous contextual factors, which often translates into a challenging experience, especially when the family member is responsible for caring for both the sick family member and other minor family members who live together and take on certain tasks in this scenario.

*[...] I even miss it... Everyone together. Everyone worked. It was another life, much happier* (Family member 6).

*[...] it’s very different. When he [father] was well, he worked, took us out, to play, to eat snacks. Ah, those things that parents do* (Young caregiver 5).

It becomes complex to try to establish a pattern of actions and reactions when caring for a loved one; such a response depends on multiple aspects, such as the degree of kinship with the sick family member, the reason that led to the care situation, the time elapsed since the discovery of the unexpected event, the bond established with the family member, and even the presence or absence of a support network.

Family members involved in this sphere of “caregiving” described their daily lives as monotonous and exhausting, with little time or energy available for personal activities and self-care, often neglecting themselves in favor of the well-being of their family member.

*My husband went through everything in that ICU. He underwent two hemodialysis sessions, had KPC, weighed 45 kilos [...]. There are times when, humanly speaking, I say, “Man, I have to live my life.” I am alive. God stopped him [husband]. But what about me? Am I going to have to stand still? [...]* (Family member 3).

*[...] it’s hard to go out for a walk. I don’t even know the last time we had the joy of going out for a walk. When one goes out, the other has to stay [...]. We live for him [son]* (Family member 5).

The routine of caring for a family member interferes with the personal lives of caregivers, limited to a routine dominated by the need to care for a loved one, often at the expense of their own needs and interests. Caregivers express a conflict between the desire to live their own lives and the need to provide constant care to their family member. In addition, the home thus takes on the connotation of an extension of work or a setting for the performance of incessant tasks.

*[...] I don’t know what it’s like to sleep properly anymore, you know? I have a hard time falling asleep because my mind never stops working. My mind doesn’t stop, you know? It’s a lot [...]. And then there’s a time when I take off, I leave here on the weekend, I stay away. I say, “No, I need to get out of here.” You know? And I breathe, I leave the house for a while* (Family member 3).

*I like to go out. I don’t like staying at home, no* (Young caregiver 7).

Taking on the role of caregiver often requires significant personal sacrifices and can lead to physical and emotional exhaustion, as noted in the discourses of young and adult caregivers. It was noted that emotional exhaustion had a negative impact on the school performance of young caregivers, a factor that can contribute to a vicious cycle of stress and low student performance, limiting their future opportunities and perpetuating conditions of family socioeconomic vulnerability, which was present in all the realities investigated in this study.

*I have anxiety attacks, so I can’t do [my schoolwork], so I have to do it at home* (Young caregiver 7).

Neglect of their own health was also observed in the responsible family members, ranging from preventive aspects to abstaining from hospitalization in the face of an already established illness. The reasons that lead to the omission of self-care are diverse, among them, the overload of responsibilities, the feeling of guilt when dedicating time to oneself, and the belief that one is the only one capable of providing the necessary care.

*As mothers, we don’t feel well at all. We are always sad. That day, I felt so unwell that I was faint for three days [...]. I went to the HU [hospital], and they kept me under observation. I almost went crazy. Why did they say I was going to be hospitalized? And if I were hospitalized there, who would clean his number two?* (Family member 4).

*So, because I care more about them. I don’t even care about myself [...]. Before, I used to have that preventive exam every year. This year I went to have it done, but I haven’t gone to get the results yet. I don’t even bother* (Family member 7).

The personality and beliefs of the family member receiving care were also observed as a challenge to the care provided by young and responsible caregivers. Resistance to accepting family care may be motivated by a feeling that privacy and freedom of choice have been violated, as it is natural for each family member to have a method of care that best suits their physical and emotional conditions.

*She [mother] complains about doing things our way, so she keeps complaining about pain, she has no patience [...]. It has to be her way, otherwise it won’t work* (Family member 7).

*[...] his [great-grandfather’s] temper has always been very strong. So, he doesn’t accept anything we say [...]. “My opinion is my opinion,” that’s how it is for him, you understand? He also swears a lot [...]. Sometimes he yells, I yell, he yells, I yell [...]. When things aren’t done the way he wants, he fights, he swears* (Young caregiver 10).

The reversal of roles in family care can lead to personal conflicts, given the effort made by caregivers to reconcile their new identity with their previous one. It was found that both younger and responsible caregivers expressed frustration at no longer having the family member they were used to. Thus, it should be noted that changes in the dynamics of the relationship between the family member being cared for and caregivers can alter the bond and trigger feelings of loss, sadness, and confusion, while caregivers need to adapt to experience their new responsibility and, at the same time, maintain a sense of themselves and their pre-existing relational bonds.

*Actually, this is very complicated for me, very difficult, because I am a woman and we miss many things. I have also had many conflicts about this, you know? Because we have physiological desires, from our bodies. I miss having a partner. I miss a person [...]. I love his [husband’s] life, but I don’t know if it’s a feeling of husband, wife, or if it’s a... I don’t know. I treat him like a son [...]. I don’t see him as my husband* (Family member 3).

*It’s like you’re taking care of a small* child (Young caregiver 5).

Most of the responsible family members demonstrated a deep spiritual communion, seeking God as a guide in decisions related to the health of the family member being cared for and in times of distress, in order to find support and comfort in the face of the situation they were experiencing.

*[...] I asked God so much [not to need a tracheostomy]. And then God calmed my heart, because I say this, it is not my will that must be done. Sometimes that was what I wanted. I wanted to hear that from God [...]. I felt that God’s answer was that, that he needed it. So, I was at peace* (Family member 2).

*[...] we seek strength in God, go to church, everything. So, it is a source of sustenance. We seek strength in God to get through this* (Family member 5).

*[...] I can also see, so as not to be an ungrateful person, speaking like this, spiritual issues... Wow, my life is perfect in these matters, you know?* (Family member 3).

Given the above, it should be noted that the experience of facing adversity encourages human beings to seek the development of their spiritual dimension, aiming to find possible answers to suffering. This process often also results in the psycho-emotional strengthening of these individuals, being a catalyst in the formation of resilience.

### Work and Care: The Thread of Survival

It was noted that those responsible expressed a desire to care for their family member, however, commitments to their employer interfered with their availability to take on full care.

*[...] in October, he [father] had a mild stroke again. So I stayed in the hospital with him for a week, and when he came home he couldn’t walk on his own anymore, and I need to work, right? To help my husband, because we have children, we have everything, we have expenses* (Family member 1).

*As long as she is here, I will take care of her. I won’t say that I will take care of her 24 hours a day, because I have to work. But as long as I am here at home, being at home, close to her... whatever she needs, I am willing* (Family member 6).

*It’s just that my mom works, right? She works, she’s a day laborer, so she works every day of the week, and my uncle works at night, so he sleeps all day too* (Young caregiver 1).

The demand for care led most of the responsible caregivers to leave their jobs, as the inflexibility of professional routines prevented them from providing adequate care. Thus, the options for viable services to balance the dual role ended up being reduced, comprising self-employed activities and informal work relationships.

*[...] that’s when I took leave from school [...]. He was hospitalized a lot, he had several bouts of pneumonia, he had pneumonia several times, the seizures he had were not controlled* (Family member 2).

*He [husband] spent five months in the ICU [...]. I worked nearby, and during visiting hours, I would go visit him and stay there with him. But when the doctor said he was going to be discharged, I had to quit my job* (Family member 3).

However, it should be noted that the need to share the care of young people did not only arise when the primary caregiver was absent. Given the demand for care, which sometimes involved complex and exhausting activities, overburdening the primary caregiver, it was necessary to call on the young caregiver to take turns providing care.

*We split into teams to take care of him. So, in the morning it’s my mom, in the afternoon it’s me. Sometimes on weekends and at night it’s me too. Like, on weekends, on Saturday I stay with him at night so my mom can rest too. Then my sister stays in the morning so my mom can rest. We take turns* (Young caregiver 9).

However, meeting the needs of a family member with a disabling condition involves more than physical sacrifice; it also implies higher financial costs, given that the treatment available to the patient through the Unified Health System (SUS) does not always meet all the family member’s needs. As a rule, some more complex cases require specific physical structures, dietary conditions, and adequate medical and hospital equipment.

*Now it’s like this, in a rush, you have to work twice as hard. Because, even though he [father] has his money, you have to buy things for him... He drinks milk, and it can’t be just any kind of milk, he has to eat a more restricted diet, he has to eat more specific foods for diabetics. So, we have to work, because his money alone isn’t enough for all that* (Family member 1).

*There are chairs that can recline a little, but they are expensive. We can’t afford to buy them. But, in a while, we’ll see, we’ll pool our money, raise some funds, find a sale so we can buy the chair* (Family member 2).

It is worth noting that the economic situation of most of the families in the study was unfavorable. In the observations made during the interviews and in the field diary notes, it was noted that most families lived in inadequate housing conditions. Thus, taking care of a loved one at home can be even more difficult for families, since poor sanitation and housing conditions can hinder treatment and delay the rehabilitation of the family member being cared for.

### A Young Hand That Cares: Considerations of The Nuclear Family

The assistance provided by young caregivers was mostly perceived as positive by the responsible family members. Some family caregivers saw young people as an extension of their own bodies, given the activities they performed in the absence of the responsible person.

*She [young caregiver] helps me a lot, her participation is very important. Very much so, because when I go out to work, she makes lunch for me. In the morning, she washes the lunch dishes [...]. The other day, I left my father [bedridden family member] having dinner, I had to go out, and she was the one who put him to bed. She helps a lot with my father in that regard* (Family member 1).

*Once, I left the house and his [son’s] feeding tube came out. Then my youngest daughter [young caregiver] taught my eldest daughter [...]. She is my right-hand* woman (Family member 2).

*My grandson helps turn him [son] in bed and put on the Uripen, which only he knows how to do. I used to put it in first, then my grandson did it once, and he did it better* (Family member 4).

However, even though those responsible recognized the essential nature of the care provided by the young person, some family members said that the care cannot be compared to the assistance they provide, as the attention given is not the same and there are some types of care that only adults can perform with excellence.

*It’s like he [young caregiver] doesn’t give the same attention that I do, but I know he takes care of him [...]. There are days when I come home tired, he’s here, before going to school, he makes lunch, gives him insulin, you know?* (Family member 6).

*They [young caregivers] do a little thing here, another there, for example, I tell them to pull the liner to line the bottom to clean him [son]. But I do everything, there’s no way a child can do what I do, because it’s cleaning poop, you have to have gloves on your hands, it’s something that’s impossible [...]. I’m an adult, I’m a grandmother, I have common sense. You have to understand that they’re children [...]. This is adult work* (Family member 4).

The responsible family members sought to protect the young caregivers, seeking help from third parties for guidance on the young person’s emotional management. In addition, they avoided assigning additional responsibilities related to the family member being cared for, in order to provide more leisure time, easing the young person’s journey in the process of caring for a family member with a disabling condition.

*Now that I can go out a little [...] depending on where I go, I take her [young caregiver-daughter] with me so she can get out of the house a little* (Family member 7).

*He [young caregiver-son] helps me a lot, but I don’t ask much of him. Because he gets very upset. He does it, but I feel like he doesn’t like it. It’s really unpleasant, isn’t it? My son was nine at the time, and they had a very close connection. They were very good friends* (Family Member 3).

Finally, it became clear that the responsible family members expressed fear of being reported, by acquaintances or by the health team itself, to the authorities responsible for guaranteeing the rights of vulnerable populations, such as children, adolescents, and the elderly. This factor acts as a strong inhibitor to sharing information about the family member’s health with relatives, friends, and neighbors, hindering the formation of an active support network.

*Some know that she [mother] is with a child [young caregiver], I won’t say everyone, but a good part of them do. The whole neighborhood here knows, and are they all really friends? Sometimes they talk to other colleagues, and you know that the conversation is not good. When you think it’s not, there’s the inspection. Then you’re not here, so are you going to defend yourself?* (Family member 6).

It was perceived that this fear is related to the phenomenon of invisibility of young caregivers in health services, given that the responsible family members try to hide the actions of this population with the family member being cared for. This situation ends up hindering the care of family members with disabling conditions, as well as making it impossible for services to intervene more assertively with family caregivers, both young and adult, who are affected by the dual role they play.

## DISCUSSION

The multifaceted adversities faced by family members and young caregivers, from the perspective of complex thinking, reveal that the care provided by these individuals precedes a process of knowledge organization, which is fundamental for the effective implementation of such practice. It is in this context that new information and challenges experienced become capable of bringing different meanings to the different situations that arise. Thus, such obstacles tend to construct a way of thinking and raise awareness of the essential aspects of family life^(8,9)^.

Facing adversity through the systemic or organizational principle links the knowledge of the parts to the knowledge of the whole, since the whole is greater than the sum of its parts. Therefore, it is essential to understand this issue as an intricate network in which multiple social, economic, cultural, educational, and relational contexts are interconnected with the phenomenon of care, which cannot be analyzed in isolation^(10)^.

The complexity of dealing with an illness in the family environment is a challenging situation for all family members. This can be attributed to several difficulties, including managing the illness, reconciling new knowledge with the existing cultural context, or, from the perspective of the family caregiver, accepting the new health condition^(11)^. In this complex perspective, the family is faced with the need to build new values and behaviors. These changes, which arise in response to the health situation, end up influencing the family’s social ideals and customs^(12)^.

Thus, such challenges cause changes at the organizational, family, and individual levels, where young people assume the role of caregiver. In this case, it is considered more common or ordinary for the younger individual, for example, a child, to care for their parents when they become older. By deviating from self-regulation, with young caregivers assuming this role prematurely, there is a break in this structure with the principle of linear causality. In this principle, the cause (care) acts on the effect (family and young caregiver) and the effect acts on the cause, highlighting the complex dynamics of providing this care^(8)^.

Family dynamics are clearly modified in a caregiving scenario, a fact that causes transformations in individual lives, resulting in ambivalent consequences as it generates physical and emotional overload and, at the same time, a feeling of reciprocity for the affection received from the family member being cared for^(1,13,14)^. The establishment of a scheme of order/disorder/organization can be perceived, marked by numerous and constant inter-feedbacks that take place in the physical, biological, and human worlds^(8)^.

From this perspective, complex thinking, when referring to the work performed by caregivers, reveals itself as a transformative element of family and social organization, which is shaped to accompany the needs of individuals who require their care^(5)^. This transformation tends to alter not only the family and its structures, but also the health system, the educational area, and, consequently, the path to be followed by these caregivers, since “being present to care” for someone changes the course of that individual’s history^(15)^.

With regard to individual transformations, the results showed that family members expressed a strengthening of faith and spirituality. It is understood that complex thinking allows us to recognize some factors of everyday knowledge, characteristic of human beings^(5)^. Thus, faith in God can be understood as a common and fundamental requirement in the conception of families and caregivers in the health-disease process, as well as in the dynamics of coping with the pathology itself^(16)^.

Family members and young caregivers also expressed dissatisfaction with the role reversal manifested after the incorporation of care. In complex thinking, based on the principle of reintroducing knowledge, role reversal operates as a mechanism for restoring the subject^(7)^. This is even more so when recognizing that care itself is a complex process in which different experiences are immersed, whose meanings and effects go beyond a relationship between the young caregiver and the person dependent on care, but which includes the whole family^(17)^. Thus, the practical and experiential knowledge acquired by caregivers is established as a process of reconstruction, both social and mental, contextualized in a specific culture and era^(8)^.

Due to the reversal of roles, some competing demands are generated, leading individuals to experience exhaustion, overload, and tension due to their multiple responsibilities. These disagreements occur due to the incompatible and conflicting needs of young people, such as reconciling care with school activities, social moments, and leisure time^(18)^.

It is clear that the care provided by young caregivers was appreciated by most of the responsible family members, who expressed the indispensability of these individuals for the optimization of family dynamics. Thus, we are both products and producers of our society, which expects linear behavior from all who constitute it^(8)^.

Young caregivers, although fundamental to this family structure and organization, often do not recognize themselves as such, since they consider caregiving to be a normal and natural activity in family reciprocity^(19)^. These young people tend not to share their experiences during caregiving, fearing reports that could result in their separation from the family^(20)^. Thus, while there is a family expectation regarding the need for care, there is also a dilemma about the implications of this situation. Given this, it is urgent that health professionals identify young caregivers in order to welcome and reassure them, offering the necessary support to the family as a whole^(21)^, with a view to improving the quality of care provided.

Finally, it is recognized that the results reflect a local reality, which limits their transferability to other contexts. It is also worth noting the lack of national and international studies on young caregivers, which has made broader comparisons difficult and highlights gaps in scientific knowledge on the subject. The complexity of family care dynamics, marked by multiple social, cultural, and relational determinants, may not have been fully captured, especially considering that family configurations, the roles assumed by young caregivers, and family decisionmaking processes vary significantly between contexts. Furthermore, although the study highlights the repercussions of caregiving on young people’s daily lives, the long-term psychological, emotional, and educational impacts were not explored in depth, nor were the cultural and social factors that influence young people’s choice to become caregivers. Finally, it should be noted that, although the study reveals important challenges experienced by this population, it did not propose to develop or test interventions, indicating the need for future research to advance the construction of support strategies and models aimed at young caregivers and their families.

## CONCLUSION

Caring for a family member with a disabling condition, especially when young caregivers are the protagonists of this situation, is a complex, multifactorial, and dynamic phenomenon, permeated by barriers, challenges, and consequences that are uniquely expressed in the trajectory of families. The findings of this study show that this trajectory is marked by ambivalent feelings, overlapping roles, and the exposure of young caregivers to situations of physical, emotional, social, and educational vulnerability.

The assistance provided by young people is mostly seen as positive by family members. However, there is a recognition that the care provided by young people cannot be compared to adult care in terms of attention and skill. Therefore, although they value the contribution of young people, family members are also aware of the complexity and challenges that this role can present for these children and adolescents.

Based on the analysis of the care process experienced by families, it was possible to construct a substantive theoretical model that explains the trajectory of families with young caregivers in the care of a family member with a disabling condition, highlighting the contexts, interactions, strategies, and consequences that permeate this phenomenon. Thus, the objective of the study was achieved by offering an in-depth and integrated theoretical understanding of this experience.

Thus, the trajectory of care provided by this population must be managed and understood by health professionals, especially those in primary health care, in a complex totality, surrounded by numerous contexts, especially by the social determinants of health.Therefore, it is imperative to move towards a complex logic, constantly (re)constructing and (re)thinking actions and strategies to reach this population in situations of socioeconomic, educational, and, above all, health vulnerability.

## Data Availability

The entire dataset supporting the results of this study was published in the article itself.
