# Supplementary material for: The complexity of caregiving amid challenges: experiences of families with young caregivers
Source: Rev Esc Enferm USP. 2026 May 11;60:e20250444. doi: 10.1590/1980-220X-REEUSP-2025-0444en (PMC13160413; doi:10.1590/1980-220X-REEUSP-2025-0444en)
Supplement: Translation

# Translation: A complexidade do cuidado em meio aos desafios: experiências das famílias com jovens cuidadores

**DOI:** 10.1590/1980-220X-REEUSP-2025-0444pt

**Authors:** Gabrieli Patricio Rissi, Bianca Machado Cruz Shibukawa, Camila Moraes Garollo Piran, Beatriz Sousa da Fonseca, Alana Vitória Escritori Cargnin, Maycon Hoffmann Cheffer, Ieda Harumi Higarashi

**Affiliations:** 1 Universidade Estadual de Maringá, Programa de Pós-Graduação em Enfermagem, Maringá, PR, Brasil.; 2 Universidade Federal de Mato Grosso do Sul, Departamento de Enfermagem, Três Lagoas, MS, Brasil.; 3 Politécnico de Leiria, Leiria, Portugal.; 4 Universidade Estadual do Centro Oeste, Departamento de Enfermagem, Guarapuava, PR, Brasil.

**Keywords:** Cuidadores, Criança, Adolescente, Sobrecarga do Cuidador, Relações Familiares

## Abstract

**Objetivo:**

Construir um modelo teórico substantivo sobre a trajetória das famílias com jovens cuidadores no processo de cuidado a um familiar com condição incapacitante.

**Metodologia:**

Estudo exploratório, qualitativo, conduzido pelos referenciais da Teoria Fundamentada nos Dados e do Pensamento Complexo. A coleta ocorreu entre agosto de 2022 e outubro de 2023, nas residências de 15 jovens e 7 familiares cuidadores. A análise seguiu três etapas: codificação aberta, axial e seletiva, com apoio do *software* ATLAS.ti^®^.

**Resultados:**

A trajetória das famílias com jovens cuidadores revelou-se um processo permeado por barreiras ao cuidado, desafios relacionais e éticos, e múltiplas repercussões para jovens e responsáveis. Evidenciaram-se barreiras associadas à indisponibilidade familiar e à sobrecarga do cuidado; desafios vinculados à resistência do familiar cuidado, ao medo de denúncia e à preocupação com a qualidade de vida do jovem cuidador; e consequências que impactam o bemestar dos jovens e de seus responsáveis.

**Conclusão:**

O cuidado protagonizado por jovens configura-se um fenômeno complexo, multifatorial e dinâmico, o qual exige atenção dos profissionais de saúde, sobretudo da atenção primária, a partir de uma compreensão integral dos contextos e determinantes sociais.

## INTRODUÇÃO

No mundo moderno, no qual todas as pessoas em boas condições de saúde e em idade produtiva são absorvidas no mercado de trabalho, a responsabilidade de cuidar não raramente acaba sendo atribuída àqueles que permanecem no domicílio. Neste contexto surge a figura dos jovens cuidadores; tal grupo é formado por indivíduos menores de 18 anos, que desempenham a função do cuidado, assistência ou apoio informal e não remunerado aos familiares^(1)^. Devido à complexidade da situação, não se sabe ao certo a prevalência de jovens cuidadores no mundo, mas estima-se um percentual entre 2 e 8% e em crescimento^(2)^.

Salienta-se ainda que os adolescentes são indivíduos em fase de maturação fisiológica, cognitiva e psicológica, não apresentando, portanto, as condições ideais para assumirem tal responsabilidade. Porém, os lares nos quais os jovens cuidadores necessitam incumbir-se do cuidado geralmente possuem uma configuração familiar na qual apenas o cuidador e o ser cuidado permanecem em casa, visto que os outros membros da família possuem outras atividades extradomiciliares^(3)^.

A escolha do jovem cuidador pela família por vezes é uma decisão natural para a manutenção do funcionamento da dinâmica familiar. Já para os jovens, existe raramente a escolha ou opção por não assumir tal incumbência; estes apenas são encarregados de realizar as atividades necessárias. Ao assumir tal compromisso, mesmo que de forma involuntária, são gerados diversos transtornos no cotidiano em decorrência do cuidado prestado^(2)^.

Embora já existam algumas evidências sobre a piora da saúde, bem-estar e do relacionamento familiar entre os jovens cuidadores, estes continuam ocultos nas agendas de discussão dos cuidados de saúde e políticas públicas da área. A transformação no âmbito dos cuidados de saúde necessita de um ambiente de aprendizado que adote uma perspectiva de complexidade^(4)^.

Nesse sentido, o pensamento complexo, que enfatiza a interconexão e interdependência de todos os fenômenos, pode explicar essa situação, não como um problema isolado, mas como parte de um sistema complexo de relações sociais e econômicas^(5)^. A compreensão da trajetória das famílias com jovens cuidadores no processo de cuidado a um familiar permitiria aos profissionais de saúde, um entendimento ampliado do fenômeno, ao mesmo tempo em que auxiliaria na divisão do fardo do cuidar^(6)^.

Portanto, o presente estudo se justifica com base na importância de buscar desvelar o papel dos jovens cuidadores e seus familiares, reconhecendo-os como seres de direito, em busca da garantia de acesso à saúde, da promoção de uma adolescência saudável e da construção de uma dinâmica familiar adequada^(2)^. Mediante o exposto, objetivou-se construir um modelo teórico substantivo sobre a trajetória das famílias com jovens cuidadores no processo de cuidado a um familiar com condição incapacitante. Diante desse contexto, definiu-se como questão de pesquisa: como se configura a trajetória das famílias com jovens cuidadores no processo de cuidado a um familiar com condição incapacitante?

## MÉTODO

### Desenho do Estudo

Pesquisa exploratória, qualitativa e que utilizou o Pensamento Complexo^(5)^. como referencial teórico e a Teoria Fundamentada nos Dados (TDF), na vertente straussiana^(7)^, como referencial metodológico. O cenário do estudo correspondeu aos domicílios de jovens e familiares cuidadores, residentes em um município localizado no noroeste do Paraná, Brasil.

### Local, População e Critérios de Seleção

As informações foram coletadas entre agosto de 2022 e outubro de 2023, a partir da utilização de questionários sociodemográficos formulados pelo próprio pesquisador e da realização de entrevistas presenciais semiestruturadas. Também foram empregadas observações não estruturadas, porém, conduzidas segundo uma metodologia padronizada, com anotações em um diário de campo, a fim de assegurar a uniformidade nos registros.

Anteriormente ao processo de aquisição dos dados os instrumentos de coleta passaram por uma validação teórica de conteúdo. Esse processo envolveu a consulta a três especialistas da área de saúde da criança e do adolescente, sendo pertinente para garantir a precisão e a relevância das ferramentas. Não foi realizado teste piloto, considerando que, em consonância com a abordagem qualitativa adotada, os instrumentos foram concebidos como flexíveis e passíveis de ajustes ao longo do processo de coleta e análise dos dados. As entrevistas foram audiogravadas e transcritas, durando em média 30 minutos. Posteriormente, os registros integrais das entrevistas foram submetidos a um processo mínimo de normalização linguística.

Dessa forma, para o primeiro grupo amostral, composto por 15 jovens cuidadores e eleito por constituir o principal objeto do estudo, os critérios de seleção foram: ser criança ou adolescente menor de 18 anos que realiza cuidados, assistência ou apoio a um familiar dependente de cuidados; realizar atividades como assistência em saúde, auxílio nas atividades diárias, assistência doméstica e/ou socioemocional; e não ser mãe ou pai responsável pelo cuidado de filhos menores.

Para a identificação dos jovens cuidadores, os Agentes Comunitários de Saúde (ACS) de cada Unidade Básica de Saúde (UBS) e Unidade de Apoio à Saúde da Família (UASF) do município foram contactados, totalizando 37 serviços de saúde. Além disso, os encarregados pelo Serviço de Atenção Domiciliar (SAD) também foram abordados. Após a identificação dos potenciais participantes pelos ACS e equipes do SAD, os pesquisadores realizaram contato direto com as famílias, apresentando os objetivos do estudo e convidando os jovens e seus responsáveis legais para participação voluntária.

Posteriormente, necessitou-se realizar uma análise mais detalhada do fenômeno em estudo com os familiares, que eram os principais responsáveis pelo cuidado e pelos jovens cuidadores. Para eles, os critérios de inclusão consistiram em: ser responsável legal pelo jovem cuidador e ser o coordenador de cuidados no âmbito familiar.

Assim, sete familiares compuseram o segundo grupo amostral, compreendendo oito dos jovens participantes, visto que dois eram irmãos e possuíam o mesmo vínculo familiar. Embora a saturação teórica tenha sido alcançada com aqueles que integraram este grupo, evidencia-se que os demais responsáveis pelos jovens cuidadores convidados a participar do estudo, não puderam integrar o grupo, em função de se recusarem a participar da pesquisa, ou devido à mudança de endereço no momento da abordagem.

### Análise dos Dados

Conforme preconiza a TDF, no princípio do método comparativo constante, a análise dos dados aconteceu conjuntamente à realização de cada entrevista. A coleta foi realizada exclusivamente pela pesquisadora principal, responsável pelo estudo no âmbito de sua tese de doutorado, a qual possuía formação e experiência prévias em pesquisa qualitativa, além de aprofundamento teórico-metodológico e revisão de literatura sobre a temática.

As entrevistas foram conduzidas a partir de roteiros semiestruturados, distintos para cada grupo amostral, considerando as especificidades dos jovens cuidadores e de seus responsáveis. As questões foram progressivamente ajustadas ao longo do processo de coleta, em consonância com a análise preliminar dos dados e com o avanço da amostragem teórica. As entrevistas foram integralmente transcritas e conferidas pela própria pesquisadora, que realizou escuta atenta dos áudios e registros em diário de campo. As transcrições não foram devolvidas aos participantes para validação, uma vez que a pesquisadora esteve presente em todas as etapas do processo.

Ressalta-se que a inclusão do segundo grupo amostral foi orientada por achados analíticos emergentes do primeiro grupo, que evidenciaram a centralidade dos pais/responsáveis, das dinâmicas familiares e das condições de trabalho no processo de cuidado, demandando aprofundamento teórico desses elementos para o desenvolvimento do modelo substantivo.

A respeito do processo analítico, realizou-se a codificação aberta, axial e seletiva. Inicialmente, os códigos abertos foram estabelecidos a partir da leitura das transcrições derivadas das entrevistas. Em seguida, na codificação axial, os códigos abertos foram agrupados por similaridade e geraram categorias e subcategorias. Por fim, na codificação seletiva, houve o aprimoramento das categorias e a estruturação do fenômeno central.

Os memorandos e diagramas foram empregados durante todo o processo analítico, especialmente no momento da codificação aberta. Objetivando otimizar e organizar a análise dos dados, utilizou-se o software ATLAS.ti^®^ como um mecanismo de sistematização dos dados.

Salienta-se também que o modelo teórico construído foi validado por quatro participantes, sendo dois jovens cuidadores e dois familiares. Nesse processo, constatou-se não haver necessidade de realizar modificações no modelo criado, uma vez que os indivíduos foram capazes de se identificar, na maioria, com o que foi proposto. Destaca-se, ainda, que este estudo seguiu todas as orientações do guia Consolidated Criteria for Reporting Qualitative Research (COREQ).

### Aspectos Éticos

O estudo foi aprovado pelo Comitê Permanente de Ética em Pesquisa com Seres Humanos, da Universidade Estadual de Maringá, sob parecer n.º 5.707.802 e CAAE 61476022.1.0000.0104. Os sujeitos expressaram concordância nos Termos de Consentimento e Assentimento Livre e Esclarecido, em duas vias de igual teor, sendo categorizados como “jovem cuidador” ou “familiar”, seguido pelo numeral representativo da ordem de realização das entrevistas. Nos casos de participação de menores de idade, o Termo de Consentimento Livre e Esclarecido foi assinado pelos responsáveis legais, e o Termo de Assentimento pelos jovens cuidadores. Ressalta-se que este estudo apresenta um recorte da teoria substantiva construída no âmbito de uma tese de doutorado intitulada “Jovens cuidadores e o papel da Atenção Primária à Saúde”, contemplando fragmentos analíticos do fenômeno central.

## RESULTADOS

### Caracterização dos Participantes

O fenômeno estudado compreendeu 22 indivíduos, os quais foram divididos em dois grupos amostrais, sendo o primeiro composto por 15 jovens cuidadores e o segundo englobando sete familiares responsáveis legalmente pelos jovens.

Os jovens cuidadores apresentaram uma variação etária entre 10 e 17 anos, com média de 13 anos. A maioria dos participantes era do sexo feminino (n = 8), da raça/cor parda (n = 10) e cursava o ensino fundamental (n = 13). O grau de parentesco predominante dos indivíduos sob cuidado foi irmão (n = 7), seguido de pais e avós/bisavós (n = 4 cada). Os motivos que desencadearam a necessidade de cuidado do familiar foram variados, envolvendo doenças crônicas, congênitas, mentais, além de acidentes e imaturidade física-cognitiva.

De forma semelhante, os familiares apresentaram uma predominância do sexo feminino (n = 5), raça/cor parda (n = 5) e faixa etária entre 40 e 49 anos (n = 3). A renda per capita prevalente foi de até ½ salário mínimo (n = 4), sendo que a maioria não se encontrava empregada no momento da pesquisa (n = 5). Sobre o estado de saúde, cinco familiares possuíam alguma condição de saúde, sendo as doenças crônicas as mais apontadas (n = 4).

### Trajetória das Famílias com Jovens Cuidadores no Processo de Cuidado A um Familiar com Condição Incapacitante

Os resultados demonstraram que a necessidade do cuidado a um membro da família, particularmente em situações de enfermidades, resultando na incapacidade de o indivíduo atender às suas necessidades básicas diárias, é frequentemente acompanhada por uma série de obstáculos. Entre eles, destacam-se a indisponibilidade familiar, ligada ao contexto financeiro e à inevitabilidade do trabalho, e a sobrecarga familiar derivada do processo patológico inerente ao indivíduo cuidado.

Esse contexto resulta em diversos desafios ao familiar responsável, dado que, além de possuir preocupações intrínsecas decorrentes da realidade vivenciada, ele também assume um papel central como referência tanto para o familiar sob seus cuidados quanto para o jovem cuidador, responsabilizando-se por todo o cenário envolvido.

Neste estudo, foi possível observar que tanto o familiar responsável quanto o jovem cuidador encontraram dificuldades em lidar com a doença/condição do familiar cuidado. Estas derivavam tanto dos desafios em desenvolver as competências necessárias à execução das atividades de cuidado, quanto no que diz respeito ao preparo emocional para lidar com as repercussões da doença na saúde física e cognitiva do ente querido. Ademais, outras dificuldades para o cuidado eram de ordem relacional, desencadeadas por posturas opositoras do familiar cuidado, seja pela negação do diagnóstico, seja pela relutância em aceitar o auxílio dos familiares na realização do autocuidado.

Os desafios enfrentados pelos familiares responsáveis relacionados à necessidade do jovem atuando no cuidado do familiar também não passaram despercebidos. Sobre isso, verificou-se a presença de preocupações do responsável em relação ao jovem cuidador; era perceptível um certo comprometimento da qualidade de vida e bem-estar do jovem, proveniente da sobrecarga com as atividades de cuidado e da ausência familiar. Ainda, foi notável a inquietude quanto ao medo de denúncias pela descoberta do jovem cuidador, pois muitos temiam o julgamento desta prática pela sociedade, como algo ilegal.

Com relação aos jovens cuidadores, por sua vez, foi evidenciada a presença de um ônus considerável, ao terem que gerenciar inúmeras preocupações, que repercutiam em consequências negativas tanto para a sua saúde física quanto mental. Dessa forma, foi possível evidenciar o comprometimento do tempo de lazer e vida social destes cuidadores, o que os levava a experimentar um estresse emocional significativo.

Ademais, notou-se que os familiares responsáveis enfrentavam importantes dilemas pessoais, ao passo que os jovens cuidadores tinham sua vida educacional comprometida em consequência do ato de cuidar. Apesar disso, os resultados indicaram que tal situação proporcionou o desenvolvimento de questões ligadas à espiritualidade dos familiares responsáveis, uma vez que muitos mencionaram o maior envolvimento com instituições religiosas e das expressões de fé em Deus.

A partir desta análise, foram elaborados fragmentos da teoria substantiva para o fenômeno central: “Trajetória das famílias com jovens cuidadores no processo de cuidado a um familiar com condição incapacitante”, representada na Figura 1.

**Figura 1:** Fragmentos da teoria substantiva para o fenômeno central do estudo.

### Retratos do Cuidado: Desafios Familiares

Evidenciou-se que tanto os familiares gerenciadores do cuidado quanto os jovens cuidadores possuem inúmeros fatores contextuais e, muitas vezes, traduz-se em uma experiência desafiadora, especialmente quando o familiar se responsabiliza pelo cuidado tanto do membro da família doente quanto por outros membros menores de idade que convivem e assumem determinadas tarefas neste cenário.

*[...] dá até saudade...Todo mundo unido. Todo mundo trabalhava. Era outra vida, bem mais alegre* (Familiar 6).

*[...] é bem diferente. Quando ele [pai] estava bom, trabalhava, levava-nos para sair, para brincar, para comer lanche. Ah, essas coisas que os pais fazem* (Jovem cuidador 5).

Torna-se complexo procurar estabelecer um padrão de ações e reações diante do cuidado de um ente querido; tal resposta depende de múltiplos aspectos, como o grau de parentesco com o familiar doente, o motivo que levou à situação de cuidado, o tempo percorrido desde a descoberta do evento inesperado, o vínculo estabelecido com o familiar e, inclusive, a presença ou não de uma rede de apoio.

Os familiares inseridos nessa esfera do “cuidar” significaram o cotidiano a partir de uma perspectiva monótona e cansativa, evidenciando pouca disponibilidade de tempo e energia para realizar programas pessoais e praticarem o autocuidado, negligenciando muitas vezes, a si, em favor do bem-estar do familiar.

*Meu marido passou por tudo naquela UTI. Passou por duas hemodiálises, teve KPC, ficou com 45 quilos [...]. Tem momentos que eu, humanamente falando, falo “cara, eu tenho que viver minha vida”. Eu estou viva. Deus parou ele [esposo]. Mas e eu? Vou ter que ficar parada?[...]* (Familiar 3).

*[...] é difícil sair para passear. Nem sei a última vez que a gente teve alegria para sair, para passear. Quando um sai, o outro tem que ficar [...]. A gente vive em função dele [filho]* (Familiar 5).

A rotina de cuidados a um familiar interfere na vida pessoal dos cuidadores, circunscrita a uma rotina dominada pela necessidade de cuidar de um ente querido, muitas vezes, às custas de suas próprias necessidades e interesses. Os cuidadores expressam um conflito entre o desejo de viver suas próprias vidas e a necessidade de prover cuidados constantes ao familiar. Além disso, o lar assume, assim, uma conotação de extensão do trabalho ou um cenário para a execução de tarefas incessantes.

*[...] eu não sei mais o que é dormir direito, sabe? Tenho muita dificuldade para pegar no sono, porque a minha cabeça não para de trabalhar. A minha mente não para, sabe? É muita coisa [...]. E aí tem um tempo que eu pego, saio daqui no final de semana, eu fico fora. Falo “Não, eu preciso sair daqui.” Entendeu? E respiro, saio um pouco de casa* (Familiar 3).

*Gosto de sair. Não gosto de ficar em casa, não* (Jovem cuidador 7).

Assumir o papel de cuidador frequentemente exige sacrifícios pessoais significativos e pode levar ao esgotamento físico e emocional, como observado nos discursos de cuidadores jovens e adultos. Notou-se que em jovens cuidadores a exaustão emocional impactou negativamente no desempenho escolar, fator que pode contribuir para um ciclo vicioso de estresse e baixo rendimento estudantil, limitando suas oportunidades futuras e perpetuando as condições de vulnerabilidade socioeconômica familiar, a qual esteve presente em todas as realidades investigadas neste estudo.

*Tenho crises de ansiedade, daí eu não consigo fazer [as tarefas] na escola, daí eu tenho que fazer em casa* (Jovem cuidador 7).

A negligência com a própria saúde também foi observada nos familiares responsáveis, variando desde aspectos preventivos até a abstenção da internação diante de uma enfermidade já estabelecida. Os motivos que levam à omissão do autocuidado são diversos, entre eles, enfatiza-se a sobrecarga de responsabilidades, o sentimento de culpa ao dedicar tempo a si, e a crença de ser o único capaz de fornecer o cuidado necessário.

*A gente, como mãe, não se sente nada bem. A gente sempre está com tristeza. Esse dia me deu um mal-estar assim que fiquei três dias desfalecida [...]. Fui lá no HU [hospital], aí eles me deixaram em observação, eu fiquei quase louca da cabeça. Por que eles falaram que eu ia ficar internada? E se eu ficasse internada lá, quem limparia o número dois dele?* (Familiar 4).

*Então, porque me preocupo mais é com eles. Eu nem ligo para mim [...]. Antes, eu fazia aquele exame preventivo todo ano. Esse ano fui lá fazer, só que eu não fui ainda pegar o resultado. Eu nem esquento a cabeça* (Familiar 7).

A personalidade e as crenças do familiar cuidado também foram observadas como um desafio à assistência dos jovens e responsáveis cuidadores. A resistência em aceitar os cuidados familiares pode ser motivada pela sensação de ter a privacidade e a capacidade de escolha violadas, por ser natural que cada familiar possua um método de cuidado que melhor se adapte às suas condições físicas e emocionais.

*Ela [mãe] reclama de fazer do nosso jeito, por isso, ela fica reclamando de dor, não tem paciência [...]. Tem que ser do jeito dela, senão não vai* (Familiar 7).

*[...] o gênio dele [bisavô] sempre foi um gênio muito forte. Então, tudo que a gente fala, ele não aceita [...]. “A minha opinião é a minha opinião”, para ele é assim, entendeu? Aí também ele é muito de xingar [...]. Às vezes ele grita, eu grito, ele grita, eu grito [...]. Quando não faz as coisas na hora que ele quer, ele briga, ele fica xingando* (Jovem cuidador 10).

A inversão de papéis no cuidado familiar pode levar a conflitos pessoais, dado o esforço empreendido pelos cuidadores no sentido de reconciliar sua nova identidade com a anterior. Constatou-se que tanto os mais jovens quanto os cuidadores responsáveis expressaram frustração por não terem mais a figura do familiar que antes era habitual. Assim, ressalta-se que as mudanças na dinâmica do relacionamento entre o familiar cuidado e cuidadores podem alterar o vínculo e desencadear sentimentos de perda, tristeza e confusão, ao passo que os cuidadores precisam se adaptar para vivenciar sua nova responsabilidade e, ao mesmo tempo, manter um senso de si e de seus vínculos relacionais pré-existentes.

*Realmente, isso para mim é muito complicado, muito difícil, porque sou mulher e a gente sente falta de muitas coisas. Eu já tive muitos conflitos também sobre isso, sabe? Porque a gente tem vontades fisiológicas, do nosso corpo. Sinto falta de uma companhia. Sinto falta de uma pessoa [...]. Amo a vida dele [esposo], mas eu não sei se é um sentimento de marido, mulher ou se é um... Não sei. Trato-o como filho [...]. Não o vejo como meu marido* (Familiar 3).

*É como se você estivesse cuidando de uma criança pequena* (Jovem cuidador 5).

A maioria dos familiares responsáveis demonstrou uma profunda comunhão espiritual, buscando a Deus como guia nas decisões relacionadas à saúde do familiar cuidado e em momentos de aflição, a fim de encontrar amparo e conforto diante da situação vivenciada.

*[...] eu pedi tanto para Deus [para não precisar de traqueostomia]. E depois Deus acalmou meu coração, porque falo assim, não é a minha vontade que tem que ser feita. Às vezes era isso que eu queria. Eu queria ouvir de Deus isso [...]. Eu senti que a resposta de Deus era essa, que ele precisava. Então, eu fiquei em paz* (Familiar 2).

*[...] a gente visa buscar força em Deus, vai para a igreja, tudo. Então, é um sustento. A gente busca força em Deus para passar por isso* (Familiar 5).

*[...] consigo perceber também, para não ser uma pessoa ingrata, falando assim, questões da parte espiritual... Nossa, minha vida é perfeita nessas questões, entende?* (Familiar 3).

Diante do exposto, salienta-se que a experiência de enfrentar adversidades estimula o ser humano a buscar o desenvolvimento de sua dimensão espiritual, visando encontrar possíveis respostas para o sofrimento. Esse processo, frequentemente, resulta também no fortalecimento psicoemocional destes indivíduos, sendo um catalisador na formação da resiliência.

### Trabalho e Cuidado: O Fio da Sobrevivência

Notou-se que os responsáveis manifestaram desejo em cuidar do familiar, contudo, os compromissos estabelecidos com o empregador interferiram na disponibilidade de assumir integralmente o cuidado.

*[...] em outubro, ele [pai] teve um leve AVC de novo. Aí fiquei uma semana internada com ele, quando ele voltou para casa já não conseguia andar mais sozinho, e eu preciso trabalhar, né? Para auxiliar o marido, porque tem as crianças, tem tudo, tem gasto* (Familiar 1).

*Enquanto ela estiver aqui, eu cuidarei dela. Não direi que cuidarei dela durante 24 horas, porque tenho que trabalhar. Mas enquanto estou aqui em casa, estando em casa, perto dela...o que precisar, eu estou disposto* (Familiar 6).

*É que minha mãe trabalha, né? Ela trabalha, faz diária, daí ela trabalha todos os dias da semana e meu tio trabalha à noite, então ele dorme o dia inteiro também* (Jovem cuidador 1).

A demanda do cuidado suscitou que grande parte dos cuidadores responsáveis deixasse seus empregos, já que a inflexibilidade das rotinas profissionais impedia o desempenho adequado do cuidado. Dessa forma, as opções de serviços viáveis para equilibrar a dupla função acabavam sendo reduzidas, compreendendo atividades autônomas e vínculos informais de trabalho.

*[...] foi aí que peguei o afastamento da escola [...]. Ele ficava muito internado, tinha várias pneumonias, ele teve várias vezes pneumonia, as convulsões que ele tinha não eram controladas* (Familiar 2).

*Ele [esposo] ficou 5 meses na UTI [...]. Eu trabalhava ali perto e, na hora da visita, eu ia visitar, ficava lá com ele. Mas quando o médico falou que ele ia ter alta, eu já tive que pedir as contas no trabalho* (Familiar 3).

Entretanto, cabe destacar que a necessidade de compartilhar o cuidado com jovens não se dava apenas nos momentos de ausência do responsável. Diante da demanda de cuidados, envolvendo por vezes a realização de atividades complexas e desgastantes, sobrecarregando o cuidador principal, levava à necessidade de acionar o jovem cuidador, para revezar o cuidado.

*A gente se separa em equipes para cuidar dele. Então, de manhã é minha mãe, à tarde sou eu. Fim de semana às vezes e à noite sou eu também. Tipo, fim de semana, sábado fico com ele à noite para minha mãe descansar também. Aí minha irmã fica de manhã para minha mãe descansar. A gente se reveza* (Jovem cuidador 9).

Contudo, considera-se que atender às necessidades de um familiar com condição incapacitante envolve mais do que o sacrifício físico, mas implica também em maiores custos financeiros, dado que apenas o tratamento disponibilizado ao paciente via Sistema Único de Saúde (SUS) nem sempre atende todas as necessidades do familiar. Por norma, alguns casos mais complexos demandam estrutura física específica, condições alimentares e equipamentos médico-hospitalares adequados.

*Agora é assim, corrido, tem que trabalhar dobrado. Porque, apesar de ele [pai] ter o seu dinheiro, é preciso comprar as coisas para ele... Ele toma leite e não pode ser qualquer tipo de leite, tem que comer uma comida mais restrita, tem que comer as coisas mais específicas para diabético. Então, a gente que tem que trabalhar, porque só o dinheiro dele não dá para tudo isso* (Familiar 1).

*Tem cadeiras que têm a possibilidade de deitar um pouquinho, mas são caras. Não temos condições de comprar. Mas, daqui a algum tempo, vamos ver, fazer uma vaquinha, arrecadar um valor, encontrar uma promoção para comprarmos a cadeira* (Familiar 2).

Torna-se válido descrever que o contexto econômico de grande parte das famílias integrantes do estudo era desfavorável. Nas observações durante as entrevistas e nas anotações do diário de campo, notou-se que a maioria das famílias vivia em condições de moradia inadequadas. Assim, assumir o cuidado de um ente querido no domicílio pode ser ainda mais difícil para as famílias, já que o saneamento básico e as condições habitacionais comprometidas podem prejudicar o tratamento e postergar a reabilitação do familiar cuidado.

### A Jovem Mão Que Cuida: Considerações da Família Nuclear

A assistência fornecida pelos jovens cuidadores foi percebida, na maioria das vezes, como positiva pelos familiares responsáveis. Alguns familiares cuidadores enxergavam os jovens como uma extensão do próprio corpo, diante das atividades que eles realizavam na ausência do responsável.

*Ela [jovem cuidadora] me ajuda muito, é muito importante a participação dela. Muito, porque assim, para eu sair para trabalhar, ela que faz o almoço para mim. Na parte da manhã, ela lava a louça do almoço [...]. Outro dia, eu deixei o pai [familiar acamado] jantando, precisei sair e foi ela que o colocou para dormir. Ela ajuda bastante nessa parte aí do pai* (Familiar 1).

*Uma vez, saí de casa e a sonda de alimentação dele [filho] saiu. Aí a minha filha mais nova [jovem cuidadora] ensinou a minha filha mais velha [...]. Ela é o meu braço direito* (Familiar 2).

*Meu neto ajuda a virá-lo [filho] na cama e colocar o Uripen, que só ele sabe. Eu colocava primeiro, então meu neto colocou uma vez, colocou melhor* (Familiar 4).

Todavia, ainda que os responsáveis reconhecessem a essencialidade do cuidado prestado pelo jovem, alguns familiares manifestaram que o cuidado não pode ser comparado com a assistência que eles fornecem, pois a atenção dispensada não é a mesma e existem cuidados que apenas adultos conseguem realizar com primazia.

*Tipo assim, ele [jovem cuidador] não dá a mesma atenção que dou, mas sei que ele cuida [...]. Tem dia que chego cansado, ele está aqui, antes de ir para a escola, ele faz almoço, aplica insulina, entendeu?* (Familiar 6).

*Eles [jovens cuidadores] fazem um negocinho aqui, outro ali, por exemplo, mando puxar o traçado para forrar por baixo para fazer a limpeza dele [filho]. Mas tudo quem faz sou eu, não tem nem como a criança fazer o que faço, porque é limpar o cocô, tem que ter a luva na mão, é uma coisa que não tem nem como [...]. Eu, que sou adulta, eu que sou avó, tenho juízo. Tem que entender que é criança [...]. Isso é serviço de adulto* (Familiar 4).

Os familiares responsáveis procuravam proteger os jovens cuidadores, buscando ajuda de terceiros para orientação sobre o manejo emocional do jovem. Ademais, eles evitavam atribuir responsabilidades adicionais relacionadas ao familiar cuidado, de maneira a buscar propiciar mais momentos de lazer, amenizando a trajetória do jovem no processo de cuidar de um familiar em condição incapacitante.

*Agora que está dando para eu sair um pouco [...] dependendo do lugar para onde eu vá, eu a levo [jovem cuidadora-filha] também para ela poder sair um pouco de casa* (Familiar 7).

*Ele [jovem cuidador-filho] me ajuda muito, mas cobro bem pouco. Porque ele fica muito sentido. Ele faz, mas sinto que ele não gosta. É realmente desagradável, não é? Meu filho, na época, tinha nove anos e eles tinham uma conexão muito grande. Eles eram muito amigos* (Familiar 3).

Por fim, evidenciou-se que os familiares responsáveis expressaram medo de serem denunciados, por conhecidos ou pela própria equipe de saúde, às instâncias responsáveis por garantir os direitos de populações vulneráveis, como crianças, adolescentes e idosos. Esse fator atua como um forte inibidor do compartilhamento de informações sobre a saúde do familiar com parentes, amigos e vizinhos, prejudicando a formação de uma rede de apoio ativa.

*Alguns sabem que ela [mãe] fica com uma criança [jovem cuidador], não falarei que todos, mas uma boa parte sim. A vizinhança toda aqui sabe e será que são todos amigos mesmo? Às vezes comenta com outro colega deles, e você sabe que a conversa não é boa. Quando você pensa que não, aí está a fiscalização. Aí você não está aqui, então você vai se defender?* (Familiar 6).

Notou-se que tal receio guarda relação com o fenômeno de invisibilidade dos jovens cuidadores diante dos serviços de saúde, dado que os familiares responsáveis tentam ocultar a atuação dessa população junto ao familiar cuidado. Tal situação acaba por dificultar a assistência ao familiar com condição incapacitante, bem como impossibilita a intervenção mais assertiva dos serviços junto aos cuidadores familiares, jovens e adultos, afetados pela dupla função que desempenham.

## DISCUSSÃO

As adversidades multifacetadas enfrentadas pelos familiares e jovens cuidadores, dentro na perspectiva do pensamento complexo, revelam que o cuidado prestado por tais indivíduos precede um processo de organização do conhecimento, fundamental para a implementação efetiva de tal prática. É nesse contexto que as novas informações e desafios vivenciados se tornam capazes de trazer significados distintos para as diferentes situações que se estabelecem. Assim, tais obstáculos tendem a construir um pensamento e sensibilizar os aspectos essenciais da vida em família^(8,9)^.

O enfrentamento das adversidades pelo princípio sistêmico ou organizacional liga o conhecimento das partes ao conhecimento do todo, pois o todo é maior que a soma das partes. Portanto, é essencial compreender tal problemática como uma rede intrincada na qual os múltiplos contextos sociais, econômicos, culturais, educacionais e relacionais, encontram-se interligados ao fenômeno do cuidado, não podendo este ser analisado de forma desagregada^(10)^.

A complexidade de lidar com uma doença no ambiente familiar é uma situação desafiadora para todos os integrantes da família. Isso pode ser atribuído a várias dificuldades, incluindo a gestão da doença, a conciliação dos novos saberes com o contexto cultural existente ou ainda, na perspectiva do familiar cuidado, em aceitar a nova condição de saúde^(11)^. Nessa perspectiva complexa, a família se vê diante da necessidade de construir novos valores e condutas. Essas mudanças, que surgem como resposta à situação de saúde, acabam por influenciar os ideais e costumes sociais da família^(12)^.

Assim, tais desafios provocam mudanças em âmbito organizacional, familiar e individual, onde os jovens assumem o papel de cuidador. Nesse caso, concebe-se como ideia mais comum ou corriqueira que o indivíduo mais jovem, por exemplo, um filho, cuide dos pais quando estes se tornem mais idosos. Ao fugir da autorregulação, ao ter jovens cuidadores assumindo precocemente este papel, admite-se haver uma ruptura dessa estrutura com o princípio da causalidade linear. Nesse princípio, a causa (cuidado) age sobre o efeito (familiar e jovem cuidador) e o efeito age sobre a causa, evidenciando a dinâmica complexa em oferecer esse cuidado^(8)^.

A dinâmica familiar é claramente modificada em um cenário de cuidado, fato que provoca transformações na vida individual, resultando em consequências ambivalentes à medida que gera sobrecarga física e emocional e, ao mesmo tempo, o sentimento de retribuição ao afeto recebido pelo familiar cuidado^(1,13,14)^. Percebe-se o estabelecimento de um esquema de ordem/desordem/organização, marcado por inúmeras e constantes inter-retroações, que têm lugar nos mundos físico, biológico e humano^(8)^.

Nessa perspectiva, o pensamento complexo, ao remeter ao trabalho desempenhado pelos cuidadores, revela-se como um elemento transformador da organização familiar e social, que se molda para acompanhar as necessidades dos indivíduos que precisam de seus cuidados^(5)^. Essa transformação tende a alterar não só o familiar e suas estruturas, como o sistema de saúde, a área educacional e, consequentemente, o caminho a ser trilhado por esses cuidadores, uma vez que o “estar presente para cuidar” de alguém muda o curso da história desse indivíduo^(15)^.

A respeito das transformações individuais, os resultados evidenciaram que os familiares expressaram um fortalecimento da fé e espiritualidade. Entende-se que o pensamento complexo permite reconhecer alguns fatores do conhecimento cotidiano, característico do ser humano^(5)^. Assim, a fé em Deus pode ser compreendida como um requisito comum e fundamental na concepção de famílias e cuidadores no processo saúde-doença, bem como na dinâmica de enfrentamento da própria patologia^(16)^.

Os familiares e jovens cuidadores também expressaram a insatisfação com a inversão de papéis manifestada após a incorporação do cuidado. No pensamento complexo, com base no princípio da reintrodução do conhecimento, a inversão de papéis opera como um mecanismo de restauração do sujeito^(7)^. Ainda mais ao reconhecer-se que o cuidado, em si, é um processo complexo no qual estão imersas diferentes experiências, cujos significados e efeitos vão além de uma relação entre jovem cuidador e pessoa dependente de cuidado, mas que inclui toda a família^(17)^. Assim, a experiência prática e vivencial adquirida pelos cuidadores se estabelece como um processo de reconstrução, tanto social quanto mental, contextualizado numa cultura e época específicas^(8)^.

Por conta da inversão de papéis, algumas demandas concorrentes são geradas, levando os indivíduos a experimentarem exaustão, sobrecarga e tensão por conta de suas múltiplas atribuições. Esses desacordos ocorrem devido às necessidades incompatíveis e conflitantes dos jovens, como conciliar o cuidado com as atividades escolares, com os momentos sociais e de lazer^(18)^.

Percebe-se que o cuidado prestado pelo jovem cuidador foi recebido com apreço pela maioria dos familiares responsáveis, os quais expressaram a indispensabilidade desses indivíduos para a otimização da dinâmica familiar. Assim, somos, ao mesmo tempo, produtos e produtores de nossa sociedade, que espera um comportamento linear de todos que a constituem^(8)^.

Os jovens cuidadores, apesar de fundamentais nesta estrutura e organização familiar, frequentemente não se reconhecem como tal, dado que consideram o cuidado como uma atividade normal e natural na reciprocidade familiar^(19)^. Esses jovens tendem a não compartilhar as experiências vivenciadas durante o cuidado, temendo denúncias que resultem em sua separação da família^(20)^. Assim, ao mesmo tempo que se observa a existência de uma expectativa familiar em relação à necessidade de cuidado, há simultaneamente um dilema sobre as implicações decorrentes dessa situação. Diante disso, é premente que os profissionais de saúde identifiquem os jovens cuidadores, de maneira a acolhê-los e tranquilizá-los, oferecendo o suporte necessário à família na totalidade^(21)^, com vistas à qualificação do cuidado prestado.

Por fim, reconhece-se que os resultados retratam uma realidade local, o que limita sua transferência para outros contextos. Destaca-se, ainda, a insuficiência de estudos nacionais e internacionais sobre jovens cuidadores, o que dificultou comparações mais amplas e evidencia lacunas no conhecimento científico acerca do tema. A complexidade da dinâmica do cuidado familiar, marcada por múltiplos determinantes sociais, culturais e relacionais, pode não ter sido plenamente captada, especialmente considerando que as configurações familiares, os papéis assumidos pelos jovens cuidadores e os processos decisórios das famílias variam significativamente entre contextos. Ademais, embora o estudo evidencie repercussões do cuidado no cotidiano dos jovens, os impactos psicológicos, emocionais e educacionais em longo prazo não foram aprofundados, assim como os fatores culturais e sociais que influenciam a escolha dos jovens como cuidadores. Por fim, ressalta-se que, apesar de o estudo desvelar importantes desafios vivenciados por essa população, não se propôs a desenvolver ou testar intervenções, indicando a necessidade de pesquisas futuras que avancem na construção de estratégias e modelos de apoio voltados a jovens cuidadores e suas famílias.

## CONCLUSÃO

Cuidar de um familiar com condição incapacitante, especialmente tendo os jovens cuidadores como protagonistas dessa (situ)ação, é um fenômeno complexo, multifatorial e dinâmico, permeado por barreiras, desafios e consequências que se expressam de maneira singular na trajetória das famílias. Os achados deste estudo evidenciam que essa trajetória é marcada por ambivalência de sentimentos, sobreposição de papéis e exposição dos jovens cuidadores a situações de vulnerabilidade física, emocional, social e educacional.

A assistência prestada pelos jovens é, na maioria dos casos, vista como positiva pelos familiares. No entanto, há um reconhecimento de que o cuidado dos jovens não pode ser comparado ao cuidado adulto em termos de atenção e habilidade. Portanto, embora valorizem a contribuição dos jovens, os familiares também estão cientes da complexidade e dos desafios que essa função pode apresentar para essas crianças e adolescentes.

A partir da análise do processo de cuidado vivenciado pelas famílias, foi possível construir um modelo teórico substantivo que explica a trajetória das famílias com jovens cuidadores no cuidado a um familiar com condição incapacitante, evidenciando os contextos, as interações, as estratégias e as consequências que permeiam esse fenômeno. Dessa forma, o objetivo do estudo foi alcançado, ao oferecer uma compreensão teórica aprofundada e integrada dessa experiência.

Assim, a trajetória do cuidado realizado por essa população deve ser gerenciada e compreendida pelos profissionais da saúde, principalmente da atenção primária à saúde, numa totalidade complexa, contornada por inúmeros contextos, especialmente pelos determinantes sociais de saúde. Portanto, é imperativo avançar para uma lógica complexa, sendo necessário (re)construir e (re)pensar constantemente as ações e estratégias para alcançar essa população em situação de vulnerabilidade socioeconômica, educacional e, principalmente, de saúde.

## Data Availability

Todo o conjunto de dados que dá suporte aos resultados deste estudo foi publicado no próprio artigo.

# Translation: La complejidad del cuidado en medio de los desafíos: experiencias de familias con jóvenes cuidadores

**DOI:** 10.1590/1980-220X-REEUSP-2025-0444es

**Authors:** Gabrieli Patricio Rissi, Bianca Machado Cruz Shibukawa, Camila Moraes Garollo Piran, Beatriz Sousa da Fonseca, Alana Vitória Escritori Cargnin, Maycon Hoffmann Cheffer, Ieda Harumi Higarashi

**Affiliations:** 1 Universidade Estadual de Maringá, Programa de Pós-Graduação em Enfermagem, Maringá, PR, Brasil.; 2 Universidade Federal de Mato Grosso do Sul, Departamento de Enfermagem, Três Lagoas, MS, Brasil.; 3 Politécnico de Leiria, Leiria, Portugal.; 4 Universidade Estadual do Centro Oeste, Departamento de Enfermagem, Guarapuava, PR, Brasil.

**Keywords:** Cuidadores, Niño, Adolescente, Carga del Cuidador, Relaciones Familiares

## Abstract

**Objetivo:**

Construir un modelo teórico sustantivo sobre la trayectoria de las familias con jóvenes cuidadores en el proceso de cuidado de un familiar con una condición incapacitante.

**Metodología:**

Estudio exploratorio, cualitativo, conducido por los referentes de la Teoría Fundamentada en los Datos y el Pensamiento Complejo. La recolección de datos se llevó a cabo entre agosto de 2022 y octubre de 2023, en los hogares de 15 jóvenes y 7 familiares cuidadores. El análisis siguió tres etapas: codificación abierta, axial y selectiva, con el apoyo del software ATLAS.ti^®^.

**Resultados:**

La trayectoria de las familias con jóvenes cuidadores reveló ser un proceso plagado de barreras para el cuidado, retos relacionales y éticos, y múltiples repercusiones para los jóvenes y sus responsables. Se evidenciaron barreras asociadas a la indisponibilidad familiar y a la sobrecarga del cuidado; desafíos vinculados a la resistencia del familiar cuidado, al miedo a la denuncia y a la preocupación por la calidad de vida del joven cuidador; y consecuencias que impactan en el bienestar de los jóvenes y de sus responsables.

**Conclusión:**

El cuidado protagonizado por jóvenes se configura como un fenómeno complejo, multifactorial y dinámico, que exige la atención de los profesionales de la salud, sobre todo de la atención primaria, a partir de una comprensión integral de los contextos y determinantes sociales.

## INTRODUCCIÓN

En el mundo moderno, en el que todas las personas en buen estado de salud y en edad productiva son absorbidas por el mercado laboral, la responsabilidad del cuidado no rara vez recae en quienes permanecen en el hogar. En este contexto surge la figura de los jóvenes cuidadores; este grupo está formado por personas menores de 18 años que desempeñan funciones de cuidado, asistencia o apoyo informal y no remunerado a sus familiares^(1)^. Debido a la complejidad de la situación, no se conoce con certeza la prevalencia de los jóvenes cuidadores en el mundo, pero se estima un porcentaje de entre el 2% y el 8%, y en aumento^(2)^.

Cabe destacar además que los adolescentes son individuos en fase de maduración fisiológica, cognitiva y psicológica, por lo que no presentan las condiciones ideales para asumir tal responsabilidad. Sin embargo, los hogares en los que los jóvenes cuidadores deben asumir el cuidado suelen tener una configuración familiar en la que solo el cuidador y la persona a cargo permanecen en casa, ya que los demás miembros de la familia tienen otras actividades fuera del hogar^(3)^.

La elección del joven cuidador por parte de la familia es a veces una decisión natural para mantener el funcionamiento de la dinámica familiar. En cuanto a los jóvenes, rara vez existe la posibilidad de elegir o la opción de no asumir tal responsabilidad; simplemente se les encarga realizar las actividades necesarias. Al asumir tal compromiso, aunque sea de manera involuntaria, se generan diversos trastornos en la vida cotidiana como consecuencia del cuidado prestado^(2)^.

Aunque ya existen algunas evidencias sobre el deterioro de la salud, el bienestar y las relaciones familiares entre los jóvenes cuidadores, estos siguen ocultos en las agendas de discusión sobre la atención de la salud y las políticas públicas del área. La transformación en el ámbito de la atención de la salud requiere un entorno de aprendizaje que adopte una perspectiva de complejidad^(4)^.

En este sentido, el pensamiento complejo, que enfatiza la interconexión y la interdependencia de todos los fenómenos, puede explicar esta situación, no como un problema aislado, sino como parte de un sistema complejo de relaciones sociales y económicas^(5)^. Comprender la trayectoria de las familias con jóvenes cuidadores en el proceso de cuidado de un familiar permitiría a los profesionales de la salud tener una visión más amplia del fenómeno, al tiempo que ayudaría a compartir la carga del cuidado^(6)^.

Por lo tanto, el presente estudio se justifica en base a la importancia de buscar desvelar el papel de los jóvenes cuidadores y sus familiares, reconociéndolos como sujetos de derechos, en busca de la garantía de acceso a la salud, la promoción de una adolescencia saludable y la construcción de una dinámica familiar adecuada^(2)^. En vista de lo anterior, se propuso construir un modelo teórico sustantivo sobre la trayectoria de las familias con jóvenes cuidadores en el proceso de cuidado de un familiar con una condición incapacitante. Ante este contexto, se definió como pregunta de investigación: ¿cómo se configura la trayectoria de las familias con jóvenes cuidadores en el proceso de cuidado de un familiar con una condición incapacitante?

## MÉTODO

### Diseño del Estudio

Investigación exploratoria y cualitativa que utilizó el Pensamiento Complejo^(5)^ como marco teórico y la Teoría Fundamentada en los Datos (TFD), en su vertiente straussiana^(7)^, como marco metodológico. El escenario del estudio correspondió a los hogares de jóvenes y familiares cuidadores, residentes en un municipio ubicado en el noroeste de Paraná, Brasil.

### Lugar, Población y Criterios de Selección

La información se recopiló entre agosto de 2022 y octubre de 2023, mediante el uso de cuestionarios sociodemográficos elaborados por el propio investigador y la realización de entrevistas presenciales semiestructuradas. También se emplearon observaciones no estructuradas, pero realizadas según una metodología estandarizada, con anotaciones en un diario de campo, a fin de garantizar la uniformidad en los registros.

Antes del proceso de recopilación de datos, los instrumentos de recolección se sometieron a una validación teórica de contenido. Este proceso implicó la consulta a tres especialistas en el área de salud infantil y adolescente, lo cual fue pertinente para garantizar la precisión y la relevancia de las herramientas. No se realizó una prueba piloto, ya que, en consonancia con el enfoque cualitativo adoptado, los instrumentos se diseñaron para ser flexibles y susceptibles de ajustes a lo largo del proceso de recolección y análisis de datos. Las entrevistas se grabaron en audio y se transcribieron, con una duración promedio de 30 minutos. Posteriormente, las grabaciones íntegras de las entrevistas se sometieron a un proceso mínimo de normalización lingüística.

De este modo, para el primer grupo de la muestra, compuesto por 15 jóvenes cuidadores y elegido por constituir el objeto principal del estudio, los criterios de selección fueron: ser un niño o adolescente menor de 18 años que brinda cuidados, asistencia o apoyo a un familiar dependiente de cuidados; realizar actividades como asistencia sanitaria, ayuda en las actividades diarias, asistencia doméstica y/o socioemocional; y no ser madre o padre responsable del cuidado de hijos menores.

Para la identificación de los jóvenes cuidadores, se contactó a los Agentes Comunitarios de Salud (ACS) de cada Unidad Básica de Salud (UBS) y Unidad de Apoyo a la Salud de la Familia (UASF) del municipio, lo que sumó un total de 37 servicios de salud. Además, también se contactó a los responsables del Servicio de Atención Domiciliaria (SAD). Tras la identificación de los posibles participantes por parte de los ACS y los equipos del SAD, los investigadores se pusieron en contacto directo con las familias, presentaron los objetivos del estudio e invitaron a los jóvenes y a sus tutores legales a participar de manera voluntaria.

Posteriormente, fue necesario realizar un análisis más detallado del fenómeno en estudio con los familiares, que eran los principales responsables del cuidado, y con los jóvenes cuidadores. Para ellos, los criterios de inclusión consistieron en: ser el tutor legal del joven cuidador y ser el coordinador de los cuidados en el ámbito familiar.

Así, siete familiares compusieron el segundo grupo de la muestra, que abarcaba a ocho de los jóvenes participantes, ya que dos eran hermanos y tenían el mismo vínculo familiar. Aunque se alcanzó la saturación teórica con quienes integraron este grupo, se observa que los demás responsables de los jóvenes cuidadores invitados a participar en el estudio no pudieron integrarse al grupo, ya sea por negarse a participar en la investigación o por haber cambiado de domicilio en el momento del acercamiento.

### Análisis de los Datos

De acuerdo con lo que recomienda la TFD, siguiendo el principio del método comparativo constante, el análisis de los datos se realizó de forma conjunta con la realización de cada entrevista. La recopilación fue llevada a cabo exclusivamente por la investigadora principal, responsable del estudio en el marco de su tesis doctoral, quien contaba con formación y experiencia previas en investigación cualitativa, además de una inmersión teórico-metodológica y una revisión de la literatura sobre el tema.

Las entrevistas se llevaron a cabo a partir de guiones semiestructurados, distintos para cada grupo de la muestra, teniendo en cuenta las especificidades de los jóvenes cuidadores y de sus responsables. Las preguntas se ajustaron progresivamente a lo largo del proceso de recopilación, en consonancia con el análisis preliminar de los datos y con el avance del muestreo teórico. Las entrevistas fueron transcritas íntegramente y revisadas por la propia investigadora, quien escuchó atentamente las grabaciones y tomó notas en un diario de campo. Las transcripciones no se devolvieron a los participantes para su validación, ya que la investigadora estuvo presente en todas las etapas del proceso.

Cabe destacar que la inclusión del segundo grupo de muestra se orientó por los hallazgos analíticos emergentes del primer grupo, que evidenciaron la centralidad de los padres/cuidadores, de las dinámicas familiares y de las condiciones de trabajo en el proceso de cuidado, lo que exigió una profundización teórica de estos elementos para el desarrollo del modelo sustantivo.

En cuanto al proceso analítico, se llevó a cabo la codificación abierta, axial y selectiva. Inicialmente, los códigos abiertos se establecieron a partir de la lectura de las transcripciones derivadas de las entrevistas. A continuación, en la codificación axial, los códigos abiertos se agruparon por similitud y generaron categorías y subcategorías. Por último, en la codificación selectiva, se perfeccionaron las categorías y se estructuró el fenómeno central.

Se utilizaron memorandos y diagramas durante todo el proceso analítico, especialmente en el momento de la codificación abierta. Con el objetivo de optimizar y organizar el análisis de los datos, se utilizó el software ATLAS.ti^®^ como mecanismo de sistematización de los datos.

Cabe destacar también que el modelo teórico construido fue validado por cuatro participantes, dos de ellos jóvenes cuidadores y dos familiares. En este proceso, se constató que no era necesario realizar modificaciones en el modelo creado, ya que la mayoría de los individuos pudieron identificarse con lo que se propuso. Cabe destacar, además, que este estudio siguió todas las orientaciones de la guía Consolidated Criteria for Reporting Qualitative Research (COREQ).

### Aspectos Éticos

El estudio fue aprobado por el Comité Permanente de Ética en Investigación con Seres Humanos de la Universidad Estatal de Maringá, bajo el dictamen n.º 5.707.802 y CAAE 61476022.1.0000.0104. Los sujetos expresaron su acuerdo en los Términos de Consentimiento y Asentimiento Libre e Informado, en dos copias de igual contenido, y fueron categorizados como “cuidador joven” o “familiar”, seguido del número representativo del orden de realización de las entrevistas. En los casos de participación de menores de edad, el Término de Consentimiento Libre e Informado fue firmado por los tutores legales, y el Término de Asentimiento por los jóvenes cuidadores. Cabe destacar que este estudio presenta un recorte de la teoría sustantiva construida en el marco de una tesis de doctorado titulada “Jóvenes cuidadores y el papel de la Atención Primaria de Salud”, contemplando fragmentos analíticos del fenómeno central.

## RESULTADOS

### Caracterización de los Participantes

El fenómeno estudiado abarcó a 22 individuos, los cuales se dividieron en dos grupos de muestra, estando el primero compuesto por 15 jóvenes cuidadores y el segundo englobando a siete familiares legalmente responsables de los jóvenes.

Los jóvenes cuidadores tenían edades comprendidas entre los 10 y los 17 años, con una media de 13 años. La mayoría de los participantes eran mujeres (n = 8), de raza/color moreno (n = 10) y cursaban la educación primaria (n = 13).

El grado de parentesco predominante de las personas a cargo fue el de hermano (n = 7), seguido de padres y abuelos/bisabuelos (n = 4 cada uno). Los motivos que desencadenaron la necesidad de cuidar al familiar fueron variados, incluyendo enfermedades crónicas, congénitas y mentales, además de accidentes e inmadurez física-cognitiva.

De manera similar, los familiares presentaban un predominio de sexo femenino (n = 5), raza/color moreno (n = 5) y rango de edad entre 40 y 49 años (n = 3). El ingreso per cápita predominante fue de hasta ½ salario mínimo (n = 4), y la mayoría no tenía empleo en el momento de la investigación (n = 5). En cuanto al estado de salud, cinco familiares padecían alguna afección, siendo las enfermedades crónicas las más señaladas (n = 4).

### Trayectoria de las Familias con Jóvenes Cuidadores en el Proceso de Cuidado de un Familiar con una Afección Incapacitante

Los resultados demostraron que la necesidad de cuidar a un miembro de la familia, particularmente en situaciones de enfermedad, que da lugar a la incapacidad del individuo para atender sus necesidades básicas diarias, suele ir acompañada de una serie de obstáculos. Entre ellos, destacan la falta de disponibilidad familiar, ligada al contexto financiero y a la inevitabilidad del trabajo, y la sobrecarga familiar derivada del proceso patológico inherente a la persona cuidada.

Este contexto plantea diversos desafíos al familiar responsable, dado que, además de tener preocupaciones intrínsecas derivadas de la realidad vivida, también asume un papel central como referencia tanto para el familiar a su cargo como para el joven cuidador, haciéndose responsable de todo el escenario involucrado.

En este estudio, se observó que tanto el familiar responsable como el joven cuidador encontraron dificultades para lidiar con la enfermedad o afección del familiar a su cargo. Estas derivaban tanto de los desafíos para desarrollar las competencias necesarias para la ejecución de las actividades de cuidado, como de la preparación emocional para lidiar con las repercusiones de la enfermedad en la salud física y cognitiva del ser querido. Además, otras dificultades para el cuidado eran de carácter relacional, desencadenadas por posturas de oposición del familiar a cargo, ya sea por la negación del diagnóstico o por la renuencia a aceptar la ayuda de los familiares en la realización del autocuidado.

Los desafíos enfrentados por los familiares responsables relacionados con la necesidad de que el joven se ocupe del cuidado del familiar tampoco pasaron desapercibidos. Al respecto, se observó la presencia de preocupaciones del responsable en relación con el joven cuidador; era perceptible un cierto deterioro de la calidad de vida y el bienestar del joven, derivado de la sobrecarga de las actividades de cuidado y de la ausencia familiar. Además, fue notable la inquietud por el temor a denuncias si se descubría al joven cuidador, ya que muchos temían que la sociedad juzgara esta práctica como algo ilegal.

En cuanto a los jóvenes cuidadores, por su parte, se evidenció la presencia de una carga considerable, al tener que gestionar innumerables preocupaciones, que repercutían en consecuencias negativas tanto para su salud física como mental. De este modo, fue posible evidenciar el compromiso del tiempo de ocio y la vida social de estos cuidadores, lo que los llevaba a experimentar un estrés emocional significativo.

Además, se observó que los familiares responsables enfrentaban importantes dilemas personales, mientras que los jóvenes cuidadores veían comprometida su vida educativa como consecuencia del acto de cuidar. A pesar de ello, los resultados indicaron que dicha situación propició el desarrollo de cuestiones relacionadas con la espiritualidad de los familiares responsables, ya que muchos mencionaron una mayor implicación con instituciones religiosas y expresiones de fe en Dios.

A partir de este análisis, se elaboraron fragmentos de la teoría sustantiva para el fenómeno central: “Trayectoria de las familias con jóvenes cuidadores en el proceso de cuidado de un familiar con una condición incapacitante”, representada en el Figura 1.

**Figura 1:** Fragmentos de la teoría sustantiva sobre el fenómeno central del estudio.

### Retratos del Cuidado: Desafíos Familiares

Se puso de manifiesto que tanto los familiares que gestionan el cuidado como los jóvenes cuidadores se enfrentan a innumerables factores contextuales y, a menudo, esto se traduce en una experiencia desafiante, especialmente cuando el familiar se hace responsable del cuidado tanto del familiar enfermo como de otros miembros menores de edad que conviven y asumen determinadas tareas en este escenario.

*[...] hasta da nostalgia... Todos unidos. Todos trabajaban. Era otra vida, mucho más alegre* (Familiar 6).

*[...] es muy diferente. Cuando él [el padre] estaba bien, trabajaba, nos llevaba a salir, a jugar, a comer algo. Ah, esas cosas que hacen los padres* (Joven cuidador 5).

Resulta complejo tratar de establecer un patrón de acciones y reacciones ante el cuidado de un ser querido; tal respuesta depende de múltiples aspectos, como el grado de parentesco con el familiar enfermo, el motivo que llevó a la situación de cuidado, el tiempo transcurrido desde el descubrimiento del evento inesperado, el vínculo establecido con el familiar e, incluso, la presencia o no de una red de apoyo.

Los familiares inmersos en esta esfera del “cuidado” describieron la vida cotidiana desde una perspectiva monótona y agotadora, poniendo de manifiesto la escasa disponibilidad de tiempo y energía para llevar a cabo actividades personales y practicar el autocuidado, descuidándose a menudo a sí mismos en favor del bienestar del familiar.

*Mi esposo pasó por todo en esa UCI. Pasó por dos hemodiálisis, le hicieron KPC, quedó con 45 kilos [...]. Hay momentos en los que, humanamente hablando, digo: “Oye, tengo que vivir mi vida”. Yo estoy viva. Dios lo detuvo [a mi esposo]. ¿Pero y yo? ¿Voy a tener que quedarme parada?[...]* (Familiar 3).

*[...] es difícil salir a pasear. Ni siquiera sé cuándo fue la última vez que tuvimos la alegría de salir, de dar un paseo. Cuando uno sale, el otro tiene que quedarse [...]. Vivimos en función de él [el hijo]* (Familiar 5).

La rutina de cuidar a un familiar interfiere en la vida personal de los cuidadores, circunscrita a una rutina dominada por la necesidad de cuidar a un ser querido, a menudo a costa de sus propias necesidades e intereses. Los cuidadores expresan un conflicto entre el deseo de vivir sus propias vidas y la necesidad de brindar cuidados constantes al familiar. Además, el hogar adquiere así una connotación de extensión del trabajo o un escenario para la ejecución de tareas incesantes.

*[...] ya no sé lo que es dormir bien, ¿sabes? Me cuesta mucho conciliar el sueño, porque mi cabeza no deja de trabajar. Mi mente no se detiene, ¿sabes? Es mucho [...]. Y entonces hay un momento en el que me voy, salgo de aquí el fin de semana, me quedo fuera. Me digo: “No, necesito salir de aquí”. ¿Entiendes? Y respiro, salgo un poco de casa* (Familiar 3).

*Me gusta salir. No me gusta quedarme en casa, no* (Joven cuidador 7).

Asumir el papel de cuidador a menudo exige sacrificios personales significativos y puede llevar al agotamiento físico y emocional, como se observa en los discursos de cuidadores jóvenes y adultos. Se observó que, en los cuidadores jóvenes, el agotamiento emocional tuvo un impacto negativo en el rendimiento escolar, factor que puede contribuir a un círculo vicioso de estrés y bajo rendimiento estudiantil, limitando sus oportunidades futuras y perpetuando las condiciones de vulnerabilidad socioeconómica familiar, que estuvo presente en todas las realidades investigadas en este estudio.

*Tengo crisis de ansiedad, por eso no puedo hacer [las tareas] en la escuela, así que tengo que hacerlas en casa* (Joven cuidador 7).

También se observó negligencia con respecto a la propia salud en los familiares responsables, que variaba desde aspectos preventivos hasta la abstención de hospitalizarse ante una enfermedad ya establecida. Los motivos que llevan a la omisión del autocuidado son diversos; entre ellos, se destacan la sobrecarga de responsabilidades, el sentimiento de culpa al dedicarse tiempo a uno mismo y la creencia de ser el único capaz de brindar el cuidado necesario.

*Una, como madre, no se siente nada bien. Siempre estamos tristes. Ese día me sentí mal y estuve tres días desmayada [...]. Fui al HU [hospital], allí me dejaron en observación, casi me volví loca. ¿Por qué dijeron que me iban a internar? ¿Y si me internaban allí, quién le limpiaría el número dos?* (Familiar 4).

*Entonces, porque me preocupo más por ellos. Ni siquiera me preocupo por mí [...]. Antes, me hacía ese examen preventivo todos los años. Este año fui a hacérmelo, pero aún no fui a recoger el resultado. Ni siquiera me preocupo* (Familiar 7).

La personalidad y las creencias del familiar a quien se cuida también se observaron como un desafío para la asistencia de los jóvenes y los cuidadores responsables. La resistencia a aceptar los cuidados familiares puede estar motivada por la sensación de que se violan la privacidad y la capacidad de elección, ya que es natural que cada familiar tenga un método de cuidado que se adapte mejor a sus condiciones físicas y emocionales.

*Ella [la madre] se queja de que lo hagamos a nuestra manera, por eso se queja de dolor, no tiene paciencia [...]. Tiene que ser a su manera, si no, no va a funcionar* (Familiar 7).

*[...] su carácter [el del bisabuelo] siempre ha sido muy fuerte. Entonces, todo lo que le decimos, no lo acepta [...]. “Mi opinión es mi opinión”, para él es así, ¿entiendes? Además, también insulta mucho [...]. A veces él grita, yo grito, él grita, yo grito [...]. Cuando no se hacen las cosas en el momento que él quiere, se pelea, se pone a insultar*(Joven cuidador 10).

La inversión de roles en el cuidado familiar puede llevar a conflictos personales, dado el esfuerzo que realizan los cuidadores para conciliar su nueva identidad con la anterior. Se observó que tanto los más jóvenes como los cuidadores responsables expresaron frustración por no tener ya la figura del familiar que antes era habitual.

Así, cabe destacar que los cambios en la dinámica de la relación entre el familiar cuidado y los cuidadores pueden alterar el vínculo y desencadenar sentimientos de pérdida, tristeza y confusión, mientras que los cuidadores necesitan adaptarse para asumir su nueva responsabilidad y, al mismo tiempo, mantener un sentido de sí mismos y de sus vínculos relacionales preexistentes.

*Realmente, esto para mí es muy complicado, muy difícil, porque soy mujer y una echa de menos muchas cosas. También he tenido muchos conflictos por esto, ¿sabes? Porque tenemos deseos fisiológicos, de nuestro cuerpo. Echo de menos la compañía. Extraño a una persona [...]. Amo la vida de él [mi esposo], pero no sé si es un sentimiento de marido, mujer o si es un... No lo sé. Lo trato como a un hijo [...]. No lo veo como a mi esposo* (Familiar 3).

*Es como si estuvieras cuidando a un niño pequeño* (Cuidador joven 5).

La mayoría de los familiares responsables demostró una profunda comunión espiritual, buscando a Dios como guía en las decisiones relacionadas con la salud del familiar al que cuidan y en momentos de aflicción, con el fin de encontrar apoyo y consuelo ante la situación que viven.

*[...] le pedí tanto a Dios [que no tuviera que necesitar una traqueotomía]. Y luego Dios calmó mi corazón, porque digo así, no es mi voluntad la que tiene que hacerse. A veces era eso lo que quería. Quería escuchar de Dios eso [...]. Sentí que la respuesta de Dios era esa, que él necesitaba. Entonces, quedé en paz* (Familiar 2).

*[...] uno busca fuerza en Dios, va a la iglesia, todo eso. Entonces, es un sustento. Buscamos fuerza en Dios para pasar por esto* (Familiar 5).

*[...] también me doy cuenta, para no ser una persona ingrata, hablando así, de cuestiones de la parte espiritual... Vaya, mi vida es perfecta en esas cuestiones, ¿entiendes?* (Familiar 3).

Ante lo expuesto, cabe destacar que la experiencia de enfrentar adversidades estimula al ser humano a buscar el desarrollo de su dimensión espiritual, con el objetivo de encontrar posibles respuestas al sufrimiento. Este proceso, con frecuencia, también resulta en el fortalecimiento psicoemocional de estos individuos, siendo un catalizador en la formación de la resiliencia.

### Trabajo y Cuidado: El Hilo de la Supervivencia

Se observó que los cuidadores manifestaron el deseo de cuidar al familiar; sin embargo, los compromisos establecidos con el empleador interfirieron en la disponibilidad para asumir el cuidado de manera integral.

*[...] en octubre, él [el padre] tuvo un leve derrame cerebral de nuevo. Entonces me quedé una semana internada con él; cuando regresó a casa ya no podía caminar solo, y yo necesito trabajar, ¿no? Para ayudar a mi esposo, porque tenemos a los niños, tenemos todo, hay gastos* (Familiar 1).

*Mientras ella esté aquí, la cuidaré. No diré que la cuidaré las 24 horas, porque tengo que trabajar. Pero mientras esté aquí en casa, estando en casa, cerca de ella... lo que necesite, estoy dispuesto* (Familiar 6).

*Es que mi mamá trabaja, ¿no? Ella trabaja, hace jornadas diarias, así que trabaja todos los días de la semana y mi tío trabaja de noche, así que él también duerme todo el día* (Joven cuidador 1).

La demanda de cuidados hizo que gran parte de los cuidadores responsables dejaran sus empleos, ya que la inflexibilidad de las rutinas profesionales impedía el desempeño adecuado de los cuidados.

De este modo, las opciones de servicios viables para equilibrar la doble función acababan reduciéndose, abarcando actividades autónomas y vínculos laborales informales.

*[...] fue ahí que me tomé una licencia de la escuela [...]. Estaba muy hospitalizado, tuvo varias neumonías, tuvo neumonía varias veces, las convulsiones que tenía no estaban controladas* (Familiar 2).

*Él [mi esposo] estuvo 5 meses en la UCI [...]. Yo trabajaba cerca y, a la hora de las visitas, iba a verlo, me quedaba allí con él. Pero cuando el médico dijo que le darían de alta, tuve que pedir mi renuncia en el trabajo* (Familiar 3).

Sin embargo, cabe destacar que la necesidad de compartir el cuidado con los jóvenes no se daba solo en los momentos de ausencia del responsable. Ante la demanda de cuidados, que a veces implicaba la realización de actividades complejas y agotadoras, sobrecargando al cuidador principal, surgía la necesidad de recurrir al joven cuidador para turnarse en el cuidado.

*Nos dividimos en equipos para cuidarlo. Entonces, por la mañana es mi mamá, por la tarde soy yo. Los fines de semana a veces y por la noche también soy yo. Por ejemplo, el fin de semana, el sábado me quedo con él por la noche para que mi mamá también descanse. Entonces mi hermana se queda por la mañana para que mi mamá descanse. Nos turnamos* (Joven cuidador 9).

Sin embargo, se considera que atender las necesidades de un familiar con una condición incapacitante implica más que el sacrificio físico, sino que también conlleva mayores costos financieros, dado que el tratamiento que se le ofrece al paciente a través del Sistema Único de Salud (SUS) no siempre satisface todas las necesidades del familiar. Por lo general, algunos casos más complejos exigen una estructura física específica, condiciones alimentarias y equipos médicohospitalarios adecuados.

*Ahora es así, a toda prisa, hay que trabajar el doble. Porque, aunque él [el padre] tiene su dinero, hay que comprarle las cosas... Toma leche y no puede ser cualquier tipo de leche, tiene que seguir una dieta más restrictiva, tiene que comer alimentos específicos para diabéticos. Entonces, nosotros tenemos que trabajar, porque solo con su dinero no alcanza para todo eso* (Familiar 1).

*Hay sillas que se pueden reclinar un poco, pero son caras. No tenemos condiciones para comprarlas. Pero, dentro de un tiempo, ya veremos, haremos una colecta, juntaremos algo de dinero, buscaremos una oferta para comprar la silla* (Familiar 2).

Cabe señalar que el contexto económico de gran parte de las familias participantes en el estudio era desfavorable. En las observaciones durante las entrevistas y en las anotaciones del diario de campo, se notó que la mayoría de las familias vivían en condiciones de vivienda inadecuadas. Así, asumir el cuidado de un ser querido en el hogar puede ser aún más difícil para las familias, ya que el saneamiento básico y las condiciones habitacionales precarias pueden perjudicar el tratamiento y retrasar la rehabilitación del familiar al que se cuida.

### La Mano Joven Que Cuida: Consideraciones de La Familia Nuclear

La asistencia brindada por los jóvenes cuidadores fue percibida, en la mayoría de los casos, como positiva por los familiares responsables. Algunos familiares cuidadores veían a los jóvenes como una extensión de su propio cuerpo, ante las actividades que realizaban en ausencia del responsable.

*Ella [la joven cuidadora] me ayuda mucho, su participación es muy importante. Mucho, porque así, para que yo salga a trabajar, ella me prepara el almuerzo. Por la mañana, lava los platos del almuerzo [...]. El otro día, dejé al papá [familiar postrado en cama] cenando, tuve que salir y fue ella quien lo acostó. Ella ayuda mucho en esa parte con el papá* (Familiar 1).

*Una vez, salí de casa y se le salió la sonda de alimentación [hijo]. Entonces mi hija menor [cuidadora joven] le enseñó a mi hija mayor [...]. Ella es mi mano derecha* (Familiar 2).

*Mi nieto ayuda a darle la vuelta [hijo] en la cama y a ponerle el Uripen, que solo él sabe cómo hacerlo. Yo se lo ponía primero, pero mi nieto se lo puso una vez y lo hizo mejor* (Familiar 4).

Sin embargo, aunque los responsables reconocían lo esencial que es el cuidado que brinda el joven, algunos familiares manifestaron que ese cuidado no se puede comparar con la asistencia que ellos brindan, ya que la atención prestada no es la misma y hay cuidados que solo los adultos pueden realizar con prioridad.

*Es como que él [el joven cuidador] no le da la misma atención que yo, pero sé que lo cuida [...]. Hay días en que llego cansado, él está aquí, antes de irse a la escuela, prepara el almuerzo, le aplica la insulina, ¿entiendes?* (Familiar 6).

*Ellos [los jóvenes cuidadores] hacen una cosita aquí, otra allá, por ejemplo, les pido que levanten la colcha para limpiar debajo de él [el hijo]. Pero todo lo hago yo, ni siquiera es posible que el niño haga lo que yo hago, porque es limpiar el popó, hay que tener el guante en la mano, es algo que ni siquiera se puede [...]. Yo, que soy adulta, que soy abuela, tengo sentido común. Hay que entender que es un niño [...]. Eso es trabajo de adultos* (Familiar 4).

Los familiares responsables buscaban proteger a los jóvenes cuidadores, solicitando ayuda de terceros para recibir orientación sobre el manejo emocional del joven. Además, evitaban asignarles responsabilidades adicionales relacionadas con el familiar a cargo, con el fin de propiciarles más momentos de ocio, aliviando así la trayectoria del joven en el proceso de cuidar a un familiar en condición de discapacidad.

*Ahora que puedo salir un poco [...] dependiendo del lugar al que vaya, la llevo [joven cuidadora-hija] también para que ella pueda salir un poco de casa* (Familiar 7).

*Él [joven cuidador-hijo] me ayuda mucho, pero le exijo muy poco. Porque se siente muy mal. Lo hace, pero siento que no le gusta. Es realmente desagradable, ¿no? Mi hijo, en ese momento, tenía nueve años y ellos tenían una conexión muy fuerte. Eran muy amigos* (Familiar 3).

Por último, se puso de manifiesto que los familiares responsables expresaron temor a ser denunciados, por conocidos o por el propio equipo de salud, ante las instancias encargadas de garantizar los derechos de las poblaciones vulnerables, como niños, adolescentes y personas mayores. Este factor actúa como un fuerte inhibidor para compartir información sobre la salud del familiar con parientes, amigos y vecinos, lo que perjudica la formación de una red de apoyo activa.

*Algunos saben que ella [la madre] se queda con un niño [cuidador joven], no diré que todos, pero sí una buena parte. Todo el vecindario aquí lo sabe y ¿acaso son todos amigos de verdad? A veces habla con otro de sus colegas, y sabes que la conversación no es buena. Cuando crees que no, ahí está la inspección. Entonces tú no estás aquí, ¿cómo te vas a defender?* (Familiar 6).

Se observó que tal temor guarda relación con el fenómeno de la invisibilidad de los jóvenes cuidadores ante los servicios de salud, dado que los familiares responsables intentan ocultar la labor de esta población junto al familiar cuidado. Tal situación termina dificultando la asistencia al familiar con condición incapacitante, así como imposibilita la intervención más asertiva de los servicios junto a los cuidadores familiares, jóvenes y adultos, afectados por la doble función que desempeñan.

## DISCUSIÓN

Las adversidades multifacéticas a las que se enfrentan los familiares y los jóvenes cuidadores, desde la perspectiva del pensamiento complejo, revelan que el cuidado prestado por estos individuos precede a un proceso de organización del conocimiento, fundamental para la implementación efectiva de dicha práctica. Es en este contexto donde la nueva información y los desafíos vividos pueden aportar significados distintos a las diferentes situaciones que se establecen. Así, dichos obstáculos tienden a construir un pensamiento y a sensibilizar sobre los aspectos esenciales de la vida familiar^(8,9)^.

El enfrentamiento de las adversidades mediante el principio sistémico u organizacional vincula el conocimiento de las partes con el conocimiento del todo, ya que el todo es mayor que la suma de las partes. Por lo tanto, es esencial comprender esta problemática como una red intrincada en la que los múltiples contextos sociales, económicos, culturales, educativos y relacionales se encuentran interconectados con el fenómeno del cuidado, el cual no puede analizarse de manera desagregada^(10)^.

La complejidad de lidiar con una enfermedad en el entorno familiar es una situación desafiante para todos los miembros de la familia. Esto puede atribuirse a varias dificultades, incluyendo el manejo de la enfermedad, la conciliación de los nuevos conocimientos con el contexto cultural existente o, desde la perspectiva del familiar que brinda cuidados, la aceptación de la nueva condición de salud^(11)^. En esta perspectiva compleja, la familia se enfrenta a la necesidad de construir nuevos valores y conductas. Estos cambios, que surgen como respuesta a la situación de salud, terminan influyendo en los ideales y costumbres sociales de la familia^(12)^.

Así, tales desafíos provocan cambios en el ámbito organizacional, familiar e individual, donde los jóvenes asumen el papel de cuidadores. En este caso, se concibe como una idea más común o habitual que el individuo más joven, por ejemplo, un hijo, cuide de los padres cuando estos se vuelven más mayores. Al alejarse de la autorregulación, al tener jóvenes cuidadores que asumen prematuramente este papel, se admite que existe una ruptura de esta estructura con el principio de causalidad lineal. En este principio, la causa (el cuidado) actúa sobre el efecto (la familia y el joven cuidador) y el efecto actúa sobre la causa, lo que pone de manifiesto la compleja dinámica que implica ofrecer ese cuidado^(8)^.

La dinámica familiar se ve claramente modificada en un escenario de cuidado, hecho que provoca transformaciones en la vida individual, lo que da lugar a consecuencias ambivalentes, ya que genera sobrecarga física y emocional y, al mismo tiempo, el sentimiento de retribuir el afecto recibido por el familiar cuidado^(1,13,14)^. Se percibe el establecimiento de un esquema de orden/desorden/organización, marcado por innumerables y constantes interrelaciones, que tienen lugar en los mundos físico, biológico y humano^(8)^.

Desde esta perspectiva, el pensamiento complejo, al referirse al trabajo realizado por los cuidadores, se revela como un elemento transformador de la organización familiar y social, que se amolda para acompañar las necesidades de los individuos que requieren sus cuidados^(5)^. Esta transformación tiende a alterar no solo la familia y sus estructuras, sino también el sistema de salud, el ámbito educativo y, en consecuencia, el camino que deben seguir estos cuidadores, ya que el “estar presente para cuidar” a alguien cambia el curso de la historia de ese individuo^(15)^.

En cuanto a las transformaciones individuales, los resultados evidenciaron que los familiares expresaron un fortalecimiento de la fe y la espiritualidad. Se entiende que el pensamiento complejo permite reconocer algunos factores del conocimiento cotidiano, característico del ser humano^(5)^. Así, la fe en Dios puede entenderse como un requisito común y fundamental en la concepción de las familias y los cuidadores en el proceso de salud-enfermedad, así como en la dinámica de afrontamiento de la propia patología^(16)^.

Los familiares y los jóvenes cuidadores también expresaron insatisfacción con la inversión de roles manifestada tras la incorporación del cuidado. En el pensamiento complejo, basado en el principio de la reintroducción del conocimiento, la inversión de roles opera como un mecanismo de restauración del sujeto^(7)^. Más aún al reconocer que el cuidado, en sí mismo, es un proceso complejo en el que se entrelazan diferentes experiencias, cuyos significados y efectos van más allá de una relación entre el joven cuidador y la persona dependiente, sino que incluye a toda la familia^(17)^. Así, la experiencia práctica y vivencial adquirida por los cuidadores se establece como un proceso de reconstrucción, tanto social como mental, contextualizado en una cultura y época específicas^(8)^.

Debido a la inversión de roles, se generan algunas exigencias concurrentes, lo que lleva a los individuos a experimentar agotamiento, sobrecarga y tensión debido a sus múltiples responsabilidades. Estos desacuerdos se producen debido a las necesidades incompatibles y conflictivas de los jóvenes, como conciliar el cuidado con las actividades escolares, con los momentos sociales y de ocio^(18)^.

Se observa que el cuidado prestado por el joven cuidador fue recibido con aprecio por la mayoría de los familiares responsables, quienes expresaron la indispensabilidad de estos individuos para la optimización de la dinámica familiar. Así, somos, al mismo tiempo, productos y productores de nuestra sociedad, que espera un comportamiento lineal de todos los que la constituyen^(8)^.

Los jóvenes cuidadores, a pesar de ser fundamentales en esta estructura y organización familiar, con frecuencia no se reconocen como tales, dado que consideran el cuidado como una actividad normal y natural en la reciprocidad familiar^(19)^. Estos jóvenes tienden a no compartir las experiencias vividas durante el cuidado, por temor a denuncias que resulten en su separación de la familia^(20)^.

Así, al mismo tiempo que se observa la existencia de una expectativa familiar respecto a la necesidad de cuidados, existe simultáneamente un dilema sobre las implicaciones derivadas de esta situación. Ante esto, es urgente que los profesionales de la salud identifiquen a los jóvenes cuidadores, a fin de acogerlos y tranquilizarlos, ofreciendo el apoyo necesario a la familia en su totalidad^(21)^, con miras a la mejora de la calidad de los cuidados prestados.

Por último, se reconoce que los resultados reflejan una realidad local, lo que limita su transferencia a otros contextos. Cabe destacar, además, la insuficiencia de estudios nacionales e internacionales sobre los jóvenes cuidadores, lo que ha dificultado comparaciones más amplias y pone de manifiesto lagunas en el conocimiento científico sobre el tema. Es posible que no se haya captado plenamente la complejidad de la dinámica del cuidado familiar, marcada por múltiples determinantes sociales, culturales y relacionales, especialmente si se tiene en cuenta que las configuraciones familiares, los roles asumidos por los jóvenes cuidadores y los procesos de toma de decisiones de las familias varían significativamente entre contextos. Además, aunque el estudio evidencia repercusiones del cuidado en la vida cotidiana de los jóvenes, no se profundizó en los impactos psicológicos, emocionales y educativos a largo plazo, así como en los factores culturales y sociales que influyen en la elección de los jóvenes como cuidadores. Por último, cabe destacar que, a pesar de que el estudio revela importantes desafíos vividos por esta población, no se propuso desarrollar ni poner a prueba intervenciones, lo que indica la necesidad de investigaciones futuras que avancen en la construcción de estrategias y modelos de apoyo dirigidos a los jóvenes cuidadores y sus familias.

## CONSIDERACIONES FINALES

Cuidar de un familiar con una condición incapacitante, especialmente cuando los jóvenes cuidadores son los protagonistas de esta situación/acción, es un fenómeno complejo, multifactorial y dinámico, impregnado de barreras, desafíos y consecuencias que se expresan de manera singular en la trayectoria de las familias. Los hallazgos de este estudio evidencian que esta trayectoria está marcada por la ambivalencia de sentimientos, la superposición de roles y la exposición de los jóvenes cuidadores a situaciones de vulnerabilidad física, emocional, social y educativa.

La asistencia prestada por los jóvenes es, en la mayoría de los casos, vista como positiva por los familiares. Sin embargo, se reconoce que el cuidado de los jóvenes no puede compararse con el cuidado de los adultos en términos de atención y habilidad. Por lo tanto, aunque valoran la contribución de los jóvenes, los familiares también son conscientes de la complejidad y los desafíos que esta función puede presentar para estos niños y adolescentes.

A partir del análisis del proceso de cuidado vivido por las familias, fue posible construir un modelo teórico sustantivo que explica la trayectoria de las familias con jóvenes cuidadores en el cuidado de un familiar con una condición incapacitante, poniendo de manifiesto los contextos, las interacciones, las estrategias y las consecuencias que impregnan este fenómeno. De esta manera, se alcanzó el objetivo del estudio, al ofrecer una comprensión teórica profunda e integrada de esta experiencia.

Por lo tanto, la trayectoria del cuidado realizado por esta población debe ser gestionada y comprendida por los profesionales de la salud, principalmente de la atención primaria, en una totalidad compleja, rodeada de innumerables contextos, especialmente por los determinantes sociales de la salud.

Por estas razones, es imperativo avanzar hacia una lógica compleja, siendo necesario (re)construir y (re)pensar constantemente las acciones y estrategias para llegar a esta población en situación de vulnerabilidad socioeconómica, educativa y, principalmente, de salud.

## Data Availability

Todo el conjunto de datos que apoya los resultados de este estudio fue publicado en el propio artículo.
